# Cell-free DNA captures tumor heterogeneity and driver alterations in rapid autopsies with pre-treated metastatic cancer

**DOI:** 10.1038/s41467-021-23394-4

**Published:** 2021-05-27

**Authors:** Bernard Pereira, Christopher T. Chen, Lipika Goyal, Charlotte Walmsley, Christopher J. Pinto, Islam Baiev, Read Allen, Laura Henderson, Supriya Saha, Stephanie Reyes, Martin S. Taylor, Donna M. Fitzgerald, Maida Williams Broudo, Avinash Sahu, Xin Gao, Wendy Winckler, A. Rose Brannon, Jeffrey A. Engelman, Rebecca Leary, James R. Stone, Catarina D. Campbell, Dejan Juric

**Affiliations:** 1 Novartis Institutes for Biomedical Research, Cambridge, MA USA; 2 Massachusetts General Hospital Cancer Center, Department of Medicine, Harvard Medical School, Boston, MA USA; 3 Department of Pathology, Massachusetts General Hospital, Boston, MA USA

**Keywords:** Breast cancer, Cancer genomics, Metastasis, Tumour heterogeneity

## Abstract

In patients with metastatic cancer, spatial heterogeneity of somatic alterations may lead to incomplete assessment of a cancer’s mutational profile when analyzing a single tumor biopsy. In this study, we perform sequencing of cell-free DNA (cfDNA) and distinct metastatic tissue samples from ten rapid autopsy cases with pre-treated metastatic cancer. We show that levels of heterogeneity in genetic biomarkers vary between patients but that gene expression signatures representative of the tumor microenvironment are more consistent. Across nine patients with plasma samples available, we are able to detect 62/62 truncal and 47/121 non-truncal point mutations in cfDNA. We observe that mutation clonality in cfDNA is correlated with the number of metastatic lesions in which the mutation is detected and use this result to derive a clonality threshold to classify truncal and non-truncal driver alterations with reasonable specificity. In contrast, mutation truncality is more often incorrectly assigned when studying single tissue samples. Our results demonstrate the utility of a single cfDNA sample relative to that of single tissue samples when treating patients with metastatic cancer.

## Abstract (web summary)

It is currently unclear if cell-free DNA samples from metastatic cancers are as informative as tissue ones for cancer profiling. Here the authors show that cell-free DNA samples from rapid autopsies capture clonal and subclonal alterations of metastatic tumours and reveal more driver alterations than single tissue samples.

## Introduction

Despite clinical advances in subsets of cancers due to modern precision therapies, initial responses to therapy are often followed by the emergence of resistance and metastasis. However, spatial heterogeneity between metastases can confound the evaluation of genetic and transcriptomic biomarkers in patients presenting with pretreated metastatic disease, as these may be present in only a subset of lesions^1–3^. In these cases, analysis of just a single tissue biopsy from a patient may mask spatial heterogeneity in actionable and resistance-associated mutations^4^. Properly accounting for spatial heterogeneity is, therefore, crucial for managing patients diagnosed with pretreated metastatic cancer.

However, sampling multiple tissue biopsies from patients is invasive and often impractical. As a potential solution, cell-free DNA (cfDNA) is increasingly being used to help determine the next line of therapy for patients with metastatic disease on treatment^5,6^. Previous studies have demonstrated that cfDNA profiling allows for detection of more heterogeneous driver alterations in a patient relative to single tissue biopsies^7–9^. However, the clinical utility of profiling cfDNA relative to sampling multiple tissue biopsies for detecting heterogeneous mutations is unclear, in part because of the difficulty of obtaining multiple tissue biopsies from patients^10–14^.

In this study, we quantify the spatial heterogeneity of genetic and transcriptomic biomarkers in tumors from ten patients from a rapid autopsy series. We observe that biomarkers predictive of response to immune checkpoint inhibitors (ICI), including tumor mutation burden and predictive gene expression signatures, are generally consistent across metastases. However, some driver mutations, including those implicated in drug resistance, are found in only subsets of lesions in a patient, and a single tissue sample is inadequate for profiling the entire genetic landscape of metastatic disease. In contrast, we are able to detect more driver alterations in cfDNA than in multiple randomly selected tissue samples for 6/9 patients. Recognizing that mutation cancer cell fraction (CCF) in tumor-derived cfDNA was correlated with the number of tissue lesions harboring a mutation, we also evaluate the possibility of using cfDNA to distinguish between truncal and non-truncal alterations in a patient, and identify a CCF threshold to classify truncal and non-truncal driver mutations with a specificity of ~0.80.

## Results

### Somatic alterations in ten rapid autopsy cases

**Fig. 1:**
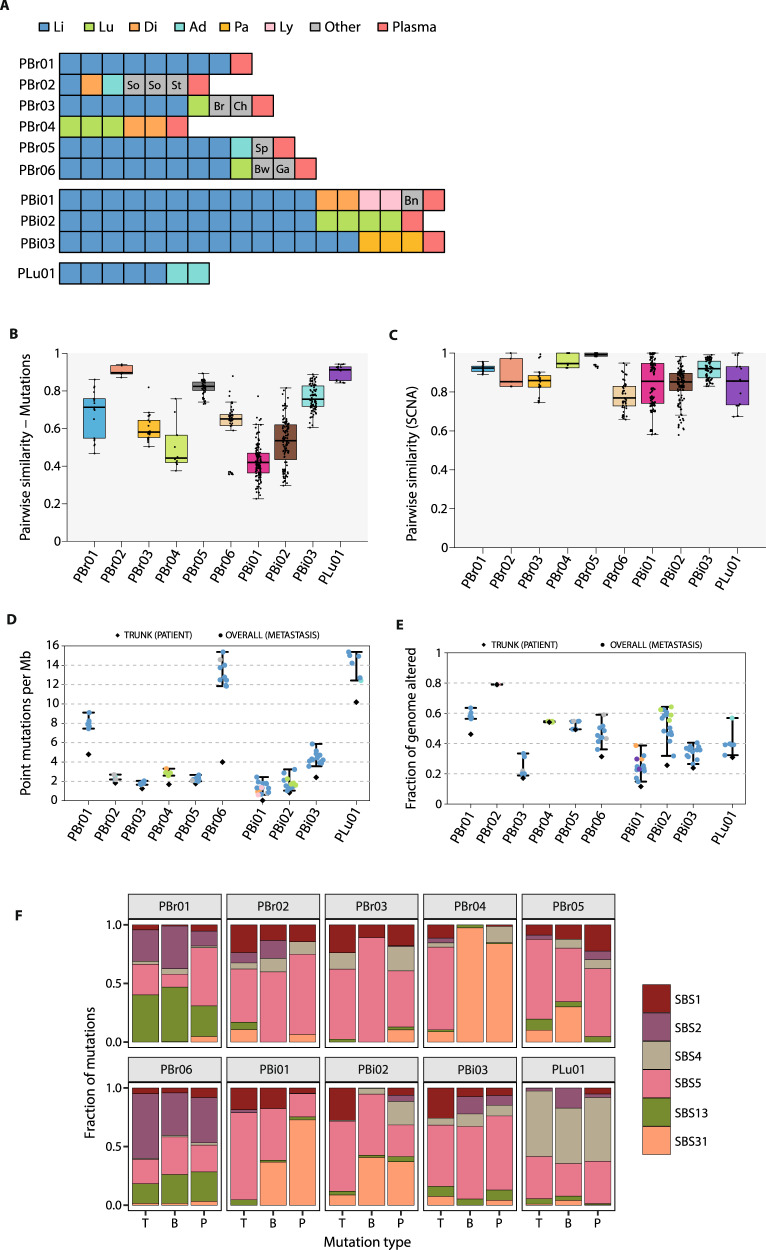
Profiling metastatic heterogeneity using rapid autopsies. **A** Summary of lesions profiled in the rapid autopsy cohort. Li liver, Lu lung, Di diaphragm, Ad adrenal gland, Pa pancreas, Ly lymph node, So soft tissue, St stomach, Br breast, Ch chest wall, Sp spleen, Bw bowel, Ga gallbladder, Bn bone. **B** For each patient, we computed the pairwise similarity based on the Jaccard index for every pair of postmortem tissue samples. Pairwise similarities, represented by the points, range from 0 (no overlap in mutations) to 1 (identical lesions). The boxplots represent the distributions of these similarities across patients. For each patient, the boxplot’s whiskers span the minimum and maximum of the distribution (excluding outliers), the box spans the lower and upper quantiles, and the center represents the median value of the distribution. **C** As for point mutations, pairwise similarity score was computed for SCNA segments. Boxplots depict the distributions as described in **B**. **D** Tumor mutation burden (TMB) was estimated by summing all classes of sSNVs and indels present at an allelic frequency of at least 0.5%. All bases in coding regions in the exome-capture panel that were covered by at least 30 reads were considered. Colored points depict TMB in individual lesions, whereas black diamonds represent the truncal mutation rate across all lesions from a patient. Error bars indicate the range of TMB estimates across lesions from a patient. **E** Copy number instability (CIN) was defined as the fraction of the genome altered by any copy number event. As for TMB, colored circular points and black diamonds represent individual lesion-specific and truncal patient-specific CIN, respectively. Error bars indicate the range of CIN estimates across lesions from a patient. **F** Mutation signatures identified in subsets of mutations across the ten patients. Bars depict the proportions of mutations within each subset that were assigned to the indicated mutation signature from the COSMIC database. Single-base substitution 1 (SBS1) and SBS5 are associated with patient age, and have been described as resembling molecular clocks. SBS2 and SBS13 are linked with AID/APOBEC activity. SBS4 has been associated with tobacco exposure and SBS31 has been linked with the mutagenic effects of platinum therapies. Source data are available for this figure.

We sequenced postmortem metastatic lesions from ten rapid autopsies, including six patients with estrogen receptor-positive (ER+) breast cancer (PBr01–Br06), three patients with cholangiocarcinoma (biliary tract cancer; PBi01–Bi03), and one patient with non-small cell lung cancer (PLu01). With the exception of PBr04, patients were treated with a median of five lines of therapy (range = 2–11) in the metastatic setting and lived with metastatic disease for a median of 35 months (range = 7–66 months; Table 1). PBr04 received neoadjuvant chemotherapy and adjuvant chemoradiation, and was diagnosed with recurrent metastatic disease ~4.5 months after the completion of her adjuvant treatment. She passed away shortly after the metastatic diagnosis without additional therapy. Complete clinical histories, including treatment details and clinical timelines, are presented in Supplementary Fig. 1. We harvested a median of nine metastatic lesions (range = 4–17) from each patient and performed both whole-exome sequencing (WES) and RNA-sequencing (RNA-seq) of the whole transcriptome on all collected lesions (Supplementary Fig. 2 and Supplementary Dataset 1). Wherever possible, we selected lesions representative of the overall spread of metastatic disease based on radiological imaging in each patient, in order to capture the full extent of spatial heterogeneity in these cancers (Fig. 1A and Supplementary Table 1). The majority of tissue samples were obtained from liver metastases (74/97). We also performed WES and RNA-seq on histologically normal tissue from each patient. For the WES data, we achieved a mean depth of coverage of between 65–207× (median = 117×) across all samples. Overall, we identified 10,111 non-silent somatic single-nucleotide variants (sSNV), 518 short insertions and deletions (indels), and somatic copy number alterations (SCNAs) across all patients (Supplementary Figs. 3 and 4, Supplementary Dataset 2, and Supplementary Dataset 3).

**Table 1:** Clinical characteristics of patients in the study.

Patient	Primary disease	Therapy lines (PRE)	Therapy lines (MET)	Survival (months after metastasis)
PBr01	ER+/PR+/HER2− breast cancer	3	5	46
PBr02	ER+/PR+/HER2− breast cancer	0	5	35
PBr03	ER+/PR+/HER2− breast cancer	0	6	64
PBr04	ER+/PR−/HER2− breast cancer	2	0^a^	0^a^
PBr05	ER+/PR+/HER2− breast cancer	3	11	66
PBr06	ER+/PR−/HER2− breast cancer	2	9	51
PBi01	Cholangiocarcinoma	0	3	9
PBi02	Cholangiocarcinoma	0	3	8
PBi03	Cholangiocarcinoma	0	3	9
PLu01	Non-small cell lung cancer	0	2	7

Treatment duration (number of months) and number of lines of therapy before (PRE) and after (MET) diagnosis of metastatic disease are shown. Complete treatment histories for all patients are presented in Supplementary Fig. 1.

^a^Passed away shortly after metastatic diagnosis; all other patients who received pre-metastatic therapy completed their therapy multiple years before metastatic diagnosis.

### Genetic biomarkers in postmortem tissue samples

To better understand the clinical implications of spatial heterogeneity, we first assessed pairwise genetic similarities across all lesions from each patient^15^. To avoid overestimating mutation heterogeneity, we rescued mutations that were not originally called in a lesion if they were identified in other lesions from the same patient and were supported by at least three reads^16,17^. We also restricted our analyses to loci where we had sufficient power in all lesions from a patient to detect a variant in at least three reads, thereby accounting for variation in sample purity and local sequencing depth (“Methods”). After using this approach, there was no association between tumor purity and the fraction of mutations private to a single tissue sample from a patient (Supplementary 5A, B).

Median pairwise similarity coefficients varied by patient as expected given the different evolutionary trajectories observed (Fig. 1B). For example, PLu01 had a highly linear evolutionary trajectory and a median similarity index of 0.91, indicating that, in general, 91% of the mutations detected in a tissue sample would have also be identified in another sample from this patient (Supplementary Fig. 6). On the other hand, the branched phylogenetic structure in PBi01 (median similarity coefficient = 0.42) would have led to underestimation of the cancer’s complete mutation profile from a single tissue sample. We observed similar results when repeating the analysis after downsampling the dataset to account for differences in the numbers of tissue samples between patients (Supplementary Fig. 5C). Similarly, although median similarity coefficients were generally higher for SCNAs (range = 0.77, PBr06–0.99, PBr05; Fig. 1C), subsets of lesions shifted the median estimates for some patients. There was no correlation between point mutation and copy number similarity coefficients (Spearman’s rho = 0.10, *p* = 0.79). Together, these observations suggest that while it may be possible to characterize a cancer’s overall copy number profile from a single sample, lower pairwise similarities in overall mutation profiles limit complete evaluation of point mutations from a single sample. In addition, outlier lesions can hinder evaluation of both SCNA and mutation profiles.

We next considered heterogeneity in biomarkers related to somatic alterations rates in the ten cases. Tumor mutation burden has been established as a clinically validated predictor of response to ICI^18^, and we therefore explored spatial heterogeneity in TMB (“Methods”). The median TMB across all lesions from each patient ranged from 1.33 (PBi01) to 14.58 (PLu01) mutations per Mb (Fig. 1D and Supplementary Dataset 1). The median absolute deviation (MAD) in TMB was relatively low across all patients (range: 0.05, PBr04–1.15, PBr06 mutations per Mb)^2^. To better interpret this level of variation, we considered a previously described scheme that classified TMB into low (1–5 mutations per Mb), intermediate (6–19 mutations per Mb), and high (20+ mutations per Mb) categories^19^. Based on this classification, no patient in the autopsy cohort had lesions that would have been classified into distinct categories. Median chromosomal instability (CIN), measured by the percentage of the genome altered by SCNA, ranged between 11.8% (PBr03)–66.8% (PBr02; Fig. 1E). Variation in CIN was also low with the highest patient MAD observed for PBr06 (MAD = 5.3%; median MAD across patients = 0.86%). Together, these data suggest that although evaluation of single biopsy may be confounded by heterogeneity in mutation identity, the observed rate of somatic alterations is generally consistent in patients presenting with pretreated metastatic disease.

### Mutation processes contributing to genetic heterogeneity

To better characterize the specific mutation processes giving rise to the observed heterogeneity, we looked for consistent patterns of base changes across the dataset (“Methods”). We compared the signatures obtained by de novo analysis with those defined by specific single-base substitution (SBS) patterns that have been previously described (Fig. 1F and Supplementary Fig. 7)^20–22^. We matched signatures observed in the autopsy cohort with those defined in the reference set on the basis of maximum cosine similarity. To better understand the chronology of mutation processes shaping the observed mutation profiles, we classified mutations as truncal, branch, or private based on their presence across each patient’s lesions: truncal alterations were identified in all metastatic lesions profiled, branch alterations were found in a subset of lesions, and private alterations were identified in only a single lesion^22^ (Supplementary Fig. 7A).

For two of the identified mutation signatures, the highest cosine similarities were observed with the previously described SBS1 (cosine similarity = 0.92) and SBS5 (cosine similarity = 0.82), both of which have been associated with patient age at diagnosis. In addition, two further observed signatures were most similar to SBS2 (cosine similarity = 0.95) and SBS13 (cosine similarity = 0.85). Both SBS2 and SBS13 have been attributed to the activity of the AID/APOBEC enzyme family and were identified in both truncal and non-truncal lesions from PBr01 and PBr06. One observed mutation signature was most similar to SBS4 (cosine similarity = 0.91), which has been associated with tobacco exposure^23^. This signature was evident in all subsets of mutations in PLu01, and its presence is consistent with the patient’s 50 pack-year smoking history. The remaining signature was most closely related to SBS31 (cosine similarity = 0.84), which has been associated with molecular damage, resulting from the treatment with platinum chemotherapeutics. We observed relatively high prevalence of SB31 mutations in PBr04, PBr05, PBi01, and PBi02. In all four patients, the prevalence of SBS31 mutations was higher in the non-truncal mutation subsets than in the truncal subset. The presence of this signature in these patients is consistent with exposure to cisplatin (Supplementary Fig. 1). Further exploration of specific treatment-related signatures confirmed the role of chemotherapy-mediated mutagenesis in shaping the mutation profiles of PBr02 and PBr03, in addition to those of the four patients above (Supplementary Fig. 7D)^23^. Overall, our observations highlight the role of cell-extrinsic processes, especially those related to treatment, in contributing to the acquisition of new mutations in this context.

We also further assessed branch mutations to determine whether they may have resulted from truncal mutations that were lost in some lesions. We observed loss of the mutant allele copy, likely via loss of heterozygosity (LOH), in at least one lesion in 7/10 patients (“Methods” and Supplementary Fig. 8A)^24^. The highest number of these mutation-loss events was observed in PBr06 (68/1473 total mutations; 4.6%). PLu01 had the highest proportion of mutation-loss events relative to total mutations (43/873; 4.9%). These observations suggest that some genetic heterogeneity may have been introduced by loss of mutations over the course of cancer progression. Notably, however, there was no evidence for mutation loss of driver mutations. Calculation of dN/dS estimates^25^ also showed that whereas positive selection of mutations was apparent for truncal mutations, there was limited evidence of positive selection in the non-truncal mutation subsets across the cohort (Supplementary Fig. 8B).

### Spatial heterogeneity in functional alterations

**Fig. 2:**
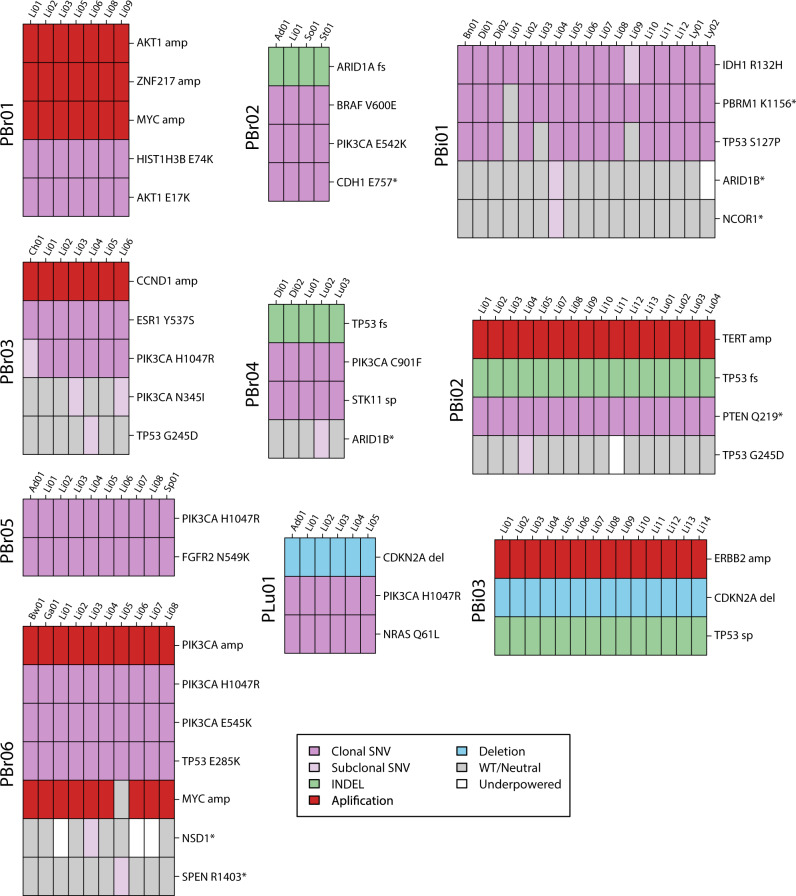
Functional alterations in ten pretreated metastatic cancer. The driver alterations (“Methods”) present in each sample are depicted by colored bars. Cancer cell fractions (CCF) of somatic SNVs are indicated by color intensity. Copy number changes depicted include high-level amplifications (amp) and deletions (loss). Gray bars indicate wild-type/copy neutral states for a particular sample. White bars indicate loci where there was not sufficient power to detect the specified mutation in a lesion (“Methods”). * nonsense SNV, fs frameshift indel, sp splice-site mutation.

We next considered heterogeneity in functional alterations across the samples from the ten patients (Fig. 2). We defined functional alterations as cancer driver and drug resistance-associated alterations based on previous studies of metastatic cancer (“Methods”)^26,27^. For each patient, we evaluated whether functional mutations were truncal or non-truncal, as defined previously. Activating truncal mutations in the PI3-K/Akt pathway were present in eight patients, including in all six breast cancer patients (Fig. 1A). Interestingly, PBr06 harbored two truncal hotspot *PIK3CA* mutations (E545K, H1047R) that both preceded high-level amplifications of *PIK3CA* in all lesions from this patient, suggesting increased dependence on *PIK3CA* signaling^28^. Copy number analysis also revealed that the high-level amplification in *AKT1* in PBr01 followed the truncal *AKT1* E17K mutation in this patient. In addition, we observed a second non-truncal *PIK3CA* (N3451) mutation in PBr03.

Across all patients, 11/42 (39.3%) of driver alterations (mutations and SCNAs) were non-truncal. Levels of heterogeneity varied by patient: all driver alterations in 5/10 patients were truncal, whereas the majority of driver alterations were non-truncal in PBi01. We observed that driver sSNVs were more than four times as likely to be clonal (i.e., found in all cells within a single lesion) than non-driver sSNVs (94.1% of driver sSNVs clonal, 19.9% of non-driver sSNVs clonal; *p* < 0.001, OR = 4.0).

We also identified mutations associated with resistance to specific therapies in some patients. PBr03 harbored a truncal *ESR1* Y537S mutation, which allows for ligand-independent ER activation, and PBr05 harbored an *FGFR2* N549K mutation^29^. Both of these mutations were truncal suggesting that the metastatic clones in these patients were derived from the expansion of the initial clones harboring these mutations. In contrast, we observed a subclonal private inactivating mutation accompanied by LOH in *SPEN* that evolved in a single liver lesion from PBr06 (PBr06-Li05). *SPEN* acts as a corepressor in the ER complex and has been associated with resistance to estrogen inhibition^30,31^.

### Heterogeneity in the metastatic tumor microenvironment

**Fig. 3:**
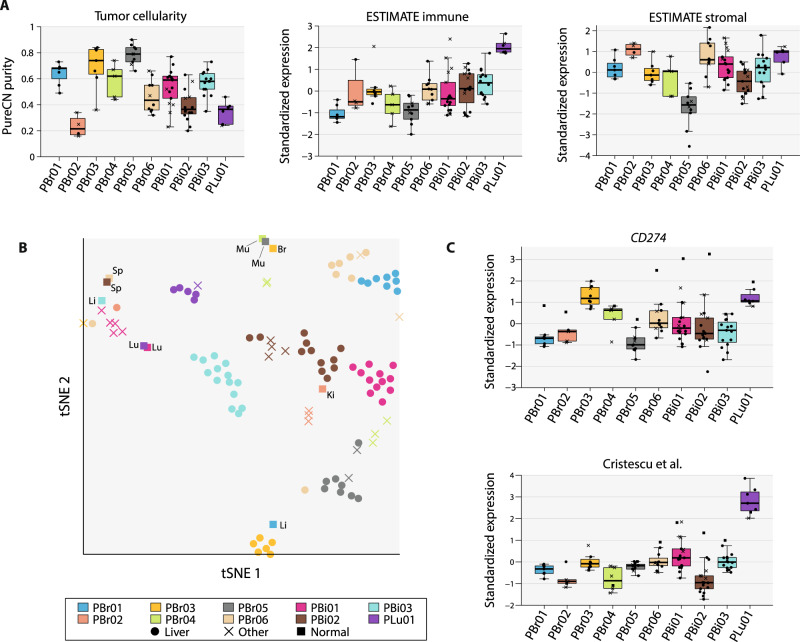
Spatial heterogeneity in the tumor microenvironment. **A** Tumor cellularity estimates (from PureCN) and measurements of overall immune and stromal signatures (from ESTIMATE) are shown for all lesions from each patient. Immune and stromal signature scores were transformed into *z*-scores across all samples and point shapes are used to differentiate between liver and non-liver lesions (circle = liver, cross = non-liver). The boxplots represent the distributions of each variable. For each patient, the boxplot’s whiskers span the minimum and maximum of the distribution, the box spans the lower and upper quantiles, and the center represents the median value of the distribution. **B** Single-sample GSEA was used to score a set of TME-related gene expression signatures and *t*-SNE was performed, using the resulting scores matrix to cluster samples by their overall TME profiles. Samples clustering together in the *t*-SNE plot suggest similarities in TME composition across the dataset. As depicted in the legend, point symbols are used to differentiate between metastases in the liver, metastases in other organs, and histologically normal samples. Anatomic sites of the normal lesions are also annotated (Ki kidney, Br breast, Lu lung, Sp spleen, Li liver, Mu skeletal muscle). **C** Expression levels of *CD274* and an ICI-predictive gene signature (Cristescu et al.) are shown. The *y*-axis indicates *z*-scores for each signature across all samples in the dataset. Point shapes are defined in the legend. The boxplots depict the variable distributions as defined in **A**. Source data are available for this figure.

We then explored spatial heterogeneity in the tumor microenvironment (TME) as this may influence response to immunomodulatory therapies. Unsupervised analysis of the of the whole transcriptome revealed that samples clustered by patient (Supplementary Fig. 9). To explore levels of immune infiltrate across samples, we first analyzed differences in tumor cellularity estimated using PureCN (Fig. 3A). In general, tissue samples from breast cancer patients had higher tumor cellularity estimates than samples from cholangiocarcinoma patients (*p* = 0.004, Wilcoxon rank-sum test). In addition, tumor cellularity MADs were low across samples from the same patient and ranged between 0.04 (PBr01)–0.18 (PBr04; median MAD = 0.09) despite being sampled from different anatomic sites. We then used ESTIMATE to quantify levels of immune and stromal expression patterns within each lesion and observed that levels of both stromal and immune-related gene sets were generally consistent across lesions from the same patient^32^. Together, these observations suggest that levels of immune infiltrate varied primarily by cancer type and by patient, but that immune infiltrate levels were more consistent across samples from a single patient.

We next considered heterogeneity in TME composition and used predefined gene expression signatures to score the expression levels of specific TME features in each sample (Supplementary Dataset 4 and Supplementary Fig. 10). We used *t*-distributed stochastic neighbor embedding (*t*-SNE) to cluster samples based on the resulting scores matrix (Fig. 3B). We observed that tumor and normal samples from the same patient clustered separately, and that the normal samples often clustered together by anatomic site. However, tumor samples from a patient clustered together suggesting that the TME profiles of lesions from the same patient were generally more similar to each other than to lesions from other patients in the context of the TME. We also computed pairwise distances in TME signature space using the scores matrix and observed that the pairwise distances between tumor samples from the same patient were significantly lower (*p* < 0.05) than pairwise distances between a patient’s tumor sample and tumor samples from all other patients for 8/10 patients (PBr02 and PBr06 not significant; Supplementary Fig. 10A). However, there was evidence of anatomic site-specific heterogeneity in two patients. In PBr02, the liver lesion had higher expression of B-cell-related signatures relative to the three lesions from other sites (Supplementary Fig. 10B). Similarly, lesions clustered by anatomic site for PBi01, driven largely by higher levels of immune infiltrate in non-liver lesions (Fig. 3A). Of note, PBi01 also had the highest proportion of non-truncal SNVs among all patients, which may have resulted in a more heterogeneous distribution of neoantigens and consequently more heterogeneous TME signatures between lesions.

We then examined variation in expression-based predictive biomarkers of response to ICI (Fig. 3C). We first analyzed expression of *CD274*, the gene that encodes PD-L1, higher expression of which has been associated with higher response rates to ICI across multiple cancer types^32^. MAD expression *z*-scores between metastases ranged from 0.13 (PLu01)–0.84 (PBi03) and was <0.5 in 7/10 patients, indicating that *CD274* expression was relatively consistent across metastases in individual patients. However, two patients (PBi01, PBi02) did have lesions that were outliers and that could, as single biopsies, influence *CD274* assessment in a patient. Finally, we analyzed the Cristescu et al. interferon gamma signature, which encompasses markers of T-cell inflammation and has been shown to predict response to anti-PD1 therapy^28,33^. This signature was correlated with *CD274* expression (*p* < 0.001, Pearson’s correlation coefficient = 0.56; Supplementary Fig. 11) and heterogeneity between lesions from a patient resembled that observed for *CD274* expression. Notably, there was high expression of this signature in PLu01 with lower scores observed across all other patients. Overall, heterogeneity in clinically validated signatures was higher between patients than between lesions from a single patient and single biopsies may therefore be suited for biomarker assessment in the context of ICI.

### Representation of somatic alterations in cfDNA

Given the genetic heterogeneity observed when comparing mutations between lesions from a patient, we next quantitatively assessed the use of liquid biopsy for characterizing mutation heterogeneity. Plasma samples for analysis of cfDNA obtained at or near time of autopsy were available for 9/10 patients (all except PLu01; Supplementary Table 1). For these samples, we performed sequencing of 566 cancer driver genes using a custom hybrid-capture assay that has previously been described (mean depth of coverage = 1007–1643×)^33^ (Supplementary Dataset 1). To maximize sensitivity within this context, we again rescued high-confidence mutations identified in the tissue samples if they were supported by three reads in cfDNA.

**Fig. 4:**
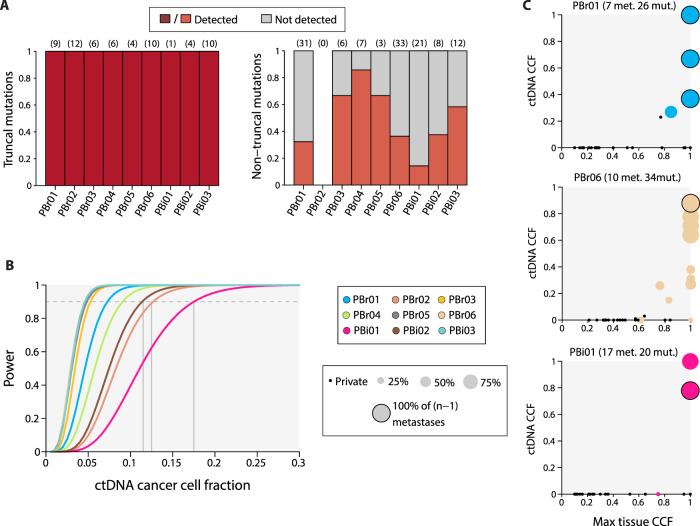
Detection of tissue mutations in cfDNA. **A** Proportions of trunk (left) and non-trunk (right) mutations, as classified using tissue samples, that were detected in cfDNA. The numbers of truncal mutations falling within the panel of 566 genes are shown above the bars. There were no branch mutations within the panel for PBr02. **B** Power curves for the detection of point mutations present in a range of ctDNA cancer cell fractions (CCF) are shown for the nine samples (colors correspond to those in Fig. 3). Power to detect point mutations depends on tumor fraction, depth of coverage, and estimated ploidy. The medium depth of coverage across all loci where a branch or private mutation was observed in tissue, but not in cfDNA was used for the calculation. The minimum ctDNA CCFs for which 90% power was achieved are indicated. **C** Representation of private (black points) and branch (colored circles) mutations in cfDNA. The CCF in which mutations were observed in ctDNA was influenced primarily by the number of tissue lesions, in which the mutation was detected (circle sizes), with the maximum CCF across all tissue samples having a lower effect. Only sSNVs were considered for analyses involving CCF estimation due to the difficulty of reliably estimating depth and VAF for somatic indels. The three samples with at least 20 branch and private mutations are shown. Met number of metastases, mut number of non-truncal mutations shown in the plot.

All truncal mutations identified in the tissue samples were also detected in cfDNA (Fig. 4A). In contrast, despite attempting to rescue mutations not originally called, only a subset of tissue branch and private mutations were detected in cfDNA, with a median of only 41.1% of the non-truncal mutations detected in cfDNA across all patients (range = 15.8–87.5%, excluding PBr02). To determine whether we were powered to detect mutations present in cfDNA, we used the estimate circulating tumor DNA (ctDNA) fraction in cfDNA to calculate the power for mutation detection across a range of ctDNA CCFs for each liquid biopsy (Supplementary Fig. 12). Power calculations were based on the median depth of coverage in tissue samples across loci, where branch and private mutations were detected in tissue but not in cfDNA. At least 90% power was available to detect mutations present in a minimum ctDNA CCF of between 0.05 (PBr05)–0.175 (PBi01) with a median minimum ctDNA CCF of 0.08 across all samples (Fig. 4B). We detected fewer than 40% of non-truncal mutations observed in tissue samples from PBr06, despite the relatively high power available in the plasma sample from this patient (90% power achieved at ctDNA CCF ≥ 0.08). In addition, non-truncal mutation detection was higher in PBr03 (4/6) than in PBi03 (7/14) even though there was 90% power for mutation detection at ctDNA CCF ≥ 0.05 in both samples. These observations suggest that low representation of branch and private mutations in cfDNA was not due solely to limited power of detection.

We reasoned that the detection of non-truncal mutations in cfDNA may be influenced by the number of metastases, in which a mutation was identified and by the clonal state of the mutation in individual tissue lesions. We focused only on sSNVs as estimates of indel variant allele fraction (VAF) can be confounded by biases in read alignment. For each sSNV, we used the maximum CCF across all patient-specific tissue lesions as a conservative measure of sSNV clonality. Multivariate analysis of the combined dataset indicated that presence in a higher number of metastases was associated with increased odds of sSNV detection in cfDNA (odds ratio, OR = 5.50, 95% confidence interval, CI = 2.62–14.56; Fig. 4C and Supplementary Fig. 13). In contrast, there was no significant association between maximum tissue CCF and sSNV detection (OR = 2.23, CI = 0.33–14.72). This result is consistent with the observation that a significantly higher proportion of branch (31/37) than of private mutations (13/80) was detected in cfDNA (*p* < 0.0001, Fisher’s exact test). Moreover, for sSNVs detected in cfDNA, mutation CCF in ctDNA was associated with the number of lesions in which the mutation was present, with an average increase in ctDNA CCF of 0.08 for every additional lesion (CI = 0.07–0.09). There was no association between clonal state and mutation CCF in ctDNA. Together, these results suggest that lesion count is a better predictor of mutation detection and mutation CCF in ctDNA than the clonal state of a mutation in any single lesion.

### Tissue samples versus cfDNA for biomarker detection

For patients with metastatic disease on treatment, molecular testing using cfDNA or tissue biopsies is often used to help determined the next line of therapy. However, the sensitivity of cfDNA relative to tissue biopsies for detecting all clinically relevant mutations is unclear. The relative utility of cfDNA to determine mutation truncality has also not been demonstrated.

**Fig. 5:**
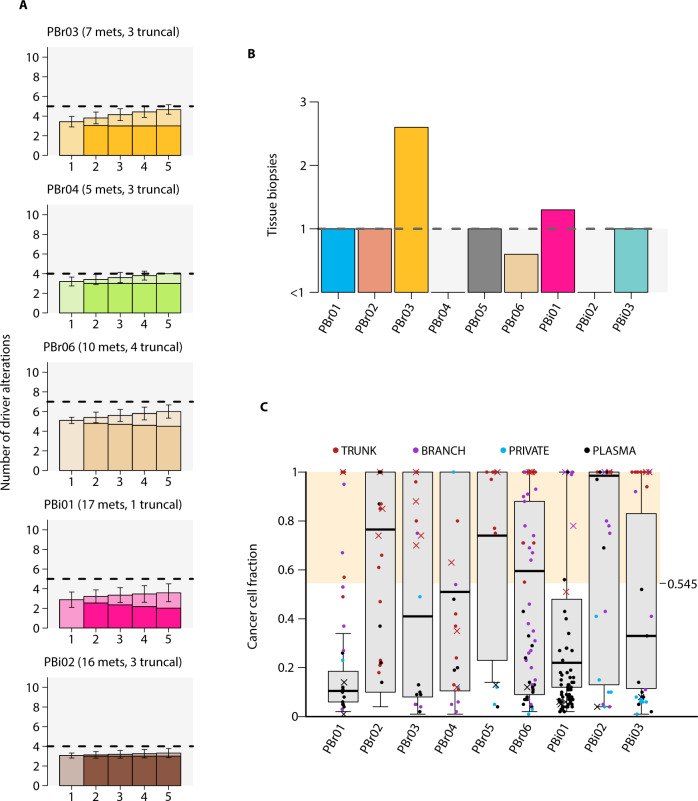
Functional alteration identification in tissue and cfDNA. **A** The average number of functional alterations (mutations and SNCAs) identified when sampling all possible combinations of one to five tissue samples from each patient are shown. For between two and five tissue samples, dark colors indicate alterations detected in lesions sampled, whereas transparent colors indicate alterations present in only a subset of the sampled lesions. The actual number of driver alterations (i.e., across all lesions) is indicated by dashed lines. The number of metastases sampled from a patient and the actual number of truncal driver alterations is noted above each plot. Errors bars indicate standard deviation in the number of detected mutations across all combinations of between one and five tissue samples. Only patients with at least one non-truncal functional alteration are shown (results for the remaining patients can be found in Supplementary Fig. 14A). **B** The number of driver alterations identified in each plasma sample compared to the number of alterations identified when sampling one to five tissue biopsies. “<1” indicates cases where more driver alterations are identified in a single tissue sample than in plasma sample. For two patients, more driver alterations are identified in the plasma sample than in a single tissue sample. **C** A threshold for distinguishing between truncal and non-truncal sSNVs was calculated using ROC analyses on the combined set of sSNVs in the nine cfDNA samples. Trunk, branch and private sSNVs are shown for each patient against the obtained threshold. Driver sSNVs are indicated by “X” points. Boxplots show the CCF distributions of sSNVs for each patient: the whiskers span the maximum and minimum CCF values, the box spans the upper and lower quartiles, and the center represents the median sSNV CCF observed for a patient.

For each patient, we first determined the mean number of driver mutations and SCNAs identified for all possible combinations of between one and five postmortem tissue samples. We focused on the five cases which had at least one non-truncal driver alteration (Fig. 5A and Supplementary Fig. 14A). Consequently, in all five cases, more than one driver alteration was missed on average when sampling a single lesion. In 4/5 cases, the number of driver alterations observed across all possible combinations of five lesions was, on average, lower than the actual number of driver alterations present in each patient. This indicates that non-truncal driver alterations would be missed in these patients even when sampling five biopsies. For PBr04, the remaining fifth case for whom five lesions were profiled in total, a mean of 3.8/4 (standard deviation, s.d. = 0.45) driver alterations were identified when sampling four biopsies, and all driver alterations were identified in only 80% of the four-lesion combinations due to the private truncating *ARID1B* mutation in this patient. Driver alteration detection in PBi01 and PBr06 was relatively poor when sampling subsets of biopsies, with a mean of 3.6/5 (PBi01; s.d. = 0.91) and 6/7 (PBr06; s.d. = 0.68) driver alterations identified when sampling five biopsies. This was largely due to private NSD1* and SPEN* mutations in this patient, as well as the absence of *MYC* amplification PBr06-Li0 (Supplementary Figs. 3 and 4). Driver alteration identification was also incomplete on average when taking five biopsies for PBi02 (mean = 3.3/4, s.d. = 0.46), which had a single private functional alteration event. The complete set of driver alterations would therefore be determined in only 22.2% and 29.4% of all combinations of five biopsies for PBr06 and PBi02, respectively (Supplementary Fig. 14B). Similarly, the complete functional alteration set would be identified in 31.3% of five-lesion samples from PBi02 and 66.7% of five-lesion samples from PBr03. When repeating the analysis using a broader list of driver mutations consisting of all non-silent mutations in a predefined list of driver genes used to study metastatic heterogeneity^22^, we identified increased levels of genetic heterogeneity, and even lower detection rate of driver sSNVs when sampling multiple biopsies (Supplementary Fig. 15).

We next explored the use of cfDNA as a tool to identify driver alterations in the nine patients with plasma samples. All functional alterations were detected from a single cfDNA sample in four patients. When functional alteration detection was incomplete in cfDNA, this was usually due to the absence of a single functional alteration. An exception was PBr06, for whom 5/7 functional alterations were detected in cfDNA (Supplementary Fig. 16). We then compared the numbers of driver alterations identified in cfDNA with those identified when sampling multiple tissue biopsies (Fig. 5B). The number of driver alterations detected in cfDNA was the same as from a single tissue sample in four cases, but was higher than the mean number of functional alterations identified across single tissue biopsies in the three cases. However, in three cases (PBr04, PBr06, PBi02), the complete set of functional alterations were detected in a single tissue sample, but not in cfDNA. Together, these analyses demonstrate that a single liquid biopsy may allow for more complete detection of driver alterations than in some combinations of multiple tissue samples, but that the presence of non-truncal alterations limits complete identification of functional alterations in some cases.

Targeted therapies may induce a more meaningful clinical response if they are directed against truncal driver alterations rather that alterations that are non-truncal. In practice, it might be assumed a mutation was truncal if it were present in all profiled biopsies. Based on this principle, we considered whether true truncal alterations could be distinguished from non-truncal alterations when randomly profiling two to five lesions (Fig. 5A). For PBr04, two tissue samples were always sufficient for accurately determining driver alteration truncality. However, in the remaining cases, some alterations were incorrectly classified as truncal in some combinations of tissue sample pairs. For example, a mean of 0.47 driver alterations were misclassified as truncal in PBr06 when sampling two lesions, with one or two additional driver alterations falsely classified as truncal in 37.8% and 4.4% of all possible pairs, respectively. A mean of 1.0 alterations were incorrectly classified as being truncal for PBi01 even when sampling five lesions (mean = 1.6 when sampling two lesions), despite the absence of any truncal alterations in this case. This observation demonstrates the potential for incorrectly classifying truncal mutations when a cancer’s evolutionary history is unknown.

Finally, we considered whether we could use cfDNA to determine mutation truncality. Given that sSNV CCF in ctDNA was correlated with the number of lesions in which the mutation occurred, we attempted to establish a CCF threshold to distinguish between truncal and non-truncal sSNVs observed in cfDNA. We performed receiver operating characteristic (ROC) analysis for the combined set of sSNVs in all cfDNA samples (area under the curve, AUC = 0.88; 95% CI = 0.84–0.92), and obtained a threshold of CCF = 0.55 (sensitivity = 0.83, specificity = 0.80) for classifying truncal sSNVs (Fig. 5C and Supplementary Fig. 17). Crucially, non-truncal driver sSNVs would have been classified as truncal using this threshold in only two cases. At least one truncal sSNVs was misclassified as non-truncal in four cases (one misclassified in PBi01, one in PBr01, five in PBr02, seven in PBr04). In contrast, we achieved a specificity of 0.65 when deriving a CCF threshold for classifying truncal mutations in a single tissue sample (sensitivity = 0.75; AUC = 0.74; CI = 0.73–0.75; Supplementary Fig. 17). Interestingly, we observed 123 sSNVs in plasma that were not identified in any of the profiled tissue samples. These sSNVs may have been present in other shedding lesions that were not profiled. However, the median CCF for these sSNVs was 0.1 (interquartile range = 0.06–0.19), and only ten of these mutations, none of which were driver alterations, were present in CCFs of at least 0.55. These results suggest that it may be possible to classify true truncal mutations from a single liquid biopsy in patients with pretreated metastatic disease.

## Discussion

In this study, we have characterized the genetic heterogeneity of ten patients with pretreated metastatic cancer by profiling multiple metastatic lesions obtained from a rapid autopsy program. We observed that functional mutations and SCNAs were most often truncal, 38.3% of functional alterations were non-truncal, and 7/10 patients had at least two non-truncal driver events. These results are in contrast to previous analyses of untreated metastatic cancers, which described notably lower driver alteration heterogeneity in that setting^22^. Together, these observations suggest that functional genetic heterogeneity may expand as individual metastatic lesions evolve over the course of treatment and progression^34^. It is possible that some subclonal mutations or copy number alterations may have not been detected at the current sequencing depth and that these may influence estimates of intratumor heterogeneity. However, we used a conservative approach by focusing on loci for which there was sufficient power to detect mutations. It is therefore also possible that our conservative estimates of intratumor genetic heterogeneity in our cohort are lower than in reality.

Global intra-patient TME composition was generally more homogeneous than inter-patient TME composition, even when considering lesions from different anatomic sites. Three TME signatures predictive of ICI response were similarly more consistent between lesions from the same patient than between patients. Tumor mutation burden estimates were similarly relatively consistent between lesions from the same patient. These results suggest that single biopsies may be informative in some patients for predicting ICI response, although the actual implications of the observed variance are unclear as clinically relevant cutoffs and associated endpoints have not been defined^35^. In addition, previous studies have suggested that significant intra-patient heterogeneity in immune checkpoint-predictive TME signatures may signal mixed responses to immunotherapy^36,37^. It is important to note that the patients in our cohort were not exposed to ICI, and that greater levels of intra-patient heterogeneity may arise in patients exposed to prior TME-modulating therapies^38–40^, or if high levels of inflammation were present at a previous stage in the cancer’s evolution^15^.

Driver alterations increase the fitness of a cancer cell and may therefore be prognostically informative or potential targets for therapeutic intervention. The importance of identifying the full spectrum driver alterations has been previously demonstrated in case studies, highlighting heterogeneous synchronous mechanisms of resistance to EGFR inhibition in metastatic lung cancers, and of resistance to PI3-K inhibition in metastatic breast cancer^41,42^. Given the observed spatial heterogeneity in driver alterations, single tissue samples were on average insufficient for identifying the full set of driver alterations in the patients from our cohort. In addition, we showed that mutations present in a single lesion can be incorrectly classified as truncal, which may be important for predicting the degree of clinical benefit from targeted^14^. It is important to note the patients received heterogeneous treatments prior to death, and that variation in levels of genetic heterogeneity may be in part attributable to different drug and resistance mechanisms.

Plasma collection is less invasive than tissue biopsies and can capture genetic alterations from multiple metastatic sites^7,43^. We found that analysis of cfDNA was superior to taking a single random tissue sample for detection of driver mutations in two patients. However, while cfDNA was highly sensitive for detecting mutations present in multiple lesions, detection of private mutations in cfDNA was limited using a validated next-generation sequencing assay. These findings are consistent with prior studies demonstrating that tumor volume is associated with mutation detection in cfDNA^44,45^ and that truncal mutations are more commonly observed in cfDNA than non-truncal mutations^45^. Detection of private mutations may improve using more recent assays although a recent study failed to detect tissue alterations in cfDNA even when sequenced to ultra-high depth^46^. In addition, recent simulations have suggested that mutations possibly restricted to single lesions may remain undetected in cfDNA due to stochastic sampling error^47^.

However, these observations offer opportunities to discriminate between truncal and non-truncal alterations using cfDNA, and we consequently attempted to establish a quantitative association between lesion count and cfDNA mutation CCF. This allowed us to derive a CCF threshold to predict whether sSNVs detected in ctDNA were truly truncal. We were able to achieve reasonable specificity, although such thresholds will vary by technical factors, cancer type and molecular background (in our dataset, all breast cancer patients had *AKT1* or *PIK3CA* mutations). More importantly, our results suggested that a better assessment of mutation truncality may be obtained from profiling cfDNA than from a single tissue lesion. We note that patients with advanced disease generally have higher cfDNA levels than patients with earlier-stage disease and that this must be considered when interpreting our findings. In addition, better understanding the mechanisms of cfDNA shedding from different lesions will also provide more context to these results.

For patients with metastatic disease, targeted drugs can be associated with high response rates and dramatic responses, but tumor heterogeneity almost inevitably results in resistance and treatment failure. Consideration of spatial genetic and transcriptomic heterogeneity may therefore result in better treatment outcomes. The results from our patient cohort provide insight into the extent of this heterogeneity, and highlight the utility of cfDNA to identify and prioritize actionable targets in pretreated metastatic cancer.

## Methods

### MGH rapid autopsy protocol

Rapid autopsies were performed by an on-call rapid autopsy team comprising of an oncologist, a pathologist, an autopsy technician, and tissue collection coordinators within 6 h of patient’s death. Fresh tissue samples were snap frozen immediately after dissection and stored at −80 °C. Blood samples were collected from the femoral vein and preserved in cfDNA BCT tubes (Streck, La Vista, NE, USA) with subsequent plasma separation, as per manufacturer’s guidelines. Separated plasma was stored at −80 °C. All specimens and clinical data were collected and analyzed in accordance with an Institutional Review Board-approved protocol (Partners Human Research Committees), to which patients provided written informed consent, and all studies were conducted in accordance with the Declaration of Helsinki. Standard hospital consent from was also signed by the health care proxy or the next of kin, as per institutional policies.

### NGS profiling of tissue and plasma samples

Simultaneous purification of DNA and RNA was performed using the Qiagen All Prep Kit (Qiagen, Wetzlar, Germany). RNA-seq libraries were prepared using an RNase H protocol^48^, and were sequenced to generate an average of 50 million reads.

The QIAmp Circulating Nucleic Acid kit (Qiagen, Wetzlar, Germany) was used for DNA extraction from frozen plasma samples. Following library construction with the Illumina TruSeq Nano DNA Library Prep kit (Illumina, San Diego, California), libraries were enriched for 566 cancer-related genes (Supplementary Table 3) using a hybrid-capture system with custom RNA baits (Agilent Technologies, Santa Clara, California). Enriched libraries were pooled and sequenced on an Illumina HiSeq 2500 machine.

### Point mutation analyses

Sequenced data were aligned with BWA-MEM^49^ and processed using the standard GATK pipeline^50^, comprising duplicate marking, indel realignment, and base quality score recalibration. Somatic SNVs were called using MuTect^51^ using default parameters for paired mutation calling. Further filtering was performed, including the removal of common polymorphisms and variants present in a panel of normal samples. A minimum depth of 30 reads at the variant loci was required in the tumor sample. Indels were called using Pindel^52^ in multisample mode for all the metastases in each patient, with additional filters similar to those used for SNVs. A minimum of three reads carrying the indel across the entire set of samples from a patient was required. To obtain a set of somatic indels, the numbers of wild-type and mutant reads in the tumor matched normal sample were compared using Fisher’s exact test, and those variants with Bonferroni-adjusted *p* < 0.01 were classified as somatic indels.

We strove to avoid overestimating genetic heterogeneity in our analyses^16^. Truncal branch and private mutations were defined using a rescue approach based on the set of high-confidence mutations identified using the above strategy. If a high-confidence mutation was observed in any single sample for a given patient, it was scored as being present in all other samples if it was supported by three reads with a minimum base quality score of 30 and a minimum mapping quality score of 60. In addition, we retained only loci for which we had 90% power to detect a variant in at least three reads, given the local depth of coverage and sample purity. As a consequence, mutations identified at a locus underpowered in any sample from a patient were excluded from analyses of heterogeneity. A similar strategy was previously used by Adalsteinsson et al.^17^. The Jaccard similarity metric was defined as the intersection of the mutations shared between two samples divided by the union of the mutations in the sample pair.

To identify functional mutations, we performed the following steps: (1) we considered only the curated set of 566 genes present on the targeted panel used for cfDNA profiling (Supplementary Table 3). (2) We compared mutations in these genes to cancer-specific driver lists from a previous study of specific driver mutations in metastatic cancers^26^. Driver gene mutations and classifications (i.e., oncogene, tumor suppressor gene) in this resource were identified on the basis of significant mutation frequencies after accounting for previously defined covariates. (3) We annotated variants in the autopsy dataset as “functional” if their mutation patterns matched those described in the cancer-specific reference list (e.g., genes with inactivating mutations that were annotated as tumor suppressor genes or genes with recurrent mutations annotated as oncogenes in the cancer-specific reference list were classified as “functional”). (4) We also annotated genes identified in the manually curated Tier 1 of the COSMIC Cancer Gene Census^27^ as functional if mutations in these genes were found in the corresponding cancer type-specific primary tumors in COSMIC. Finally, we filtered out mutations in putative driver genes that are likely due to mis-annotation. For example, there was a high frequency of mutations in KMT2C across all samples, but this may be a consequence of read misalignment due to high levels of sequence homology in the genome^53^.

Tumor mutation burden was estimated using the entire set of point mutations in coding regions observed for each sample. Bases covered by fewer than 30 reads were excluded from the calculation, and only mutations with VAF > 0.01 were considered.

A mutation-loss event was defined as the presence of mutations in some lesions from a patient, but absent in at least one lesion with loss of the minor allele (LOH), as determined by PureCN. For each locus with evidence of mutation loss, the mutation had to be observed in the absence of LOH in at least one lesion. If neither mutation nor LOH was observed for a locus in a single lesion, the locus was removed from consideration. This conservative rule was used to avoid calling mutation-loss events at LOH, where loss of a wild-type allele may have occurred. There was no evidence of mutation loss occurring by homozygous deletion.

### Mutation signature analyses

De novo mutation signature extraction was performed using the MutationalPatterns package^54^, which uses NMF deconvolution to identify consistent patterns across the dataset. The factorization rank (number of clusters) was initially determined using changes in the cophenetic correlation coefficient, as suggested. The extracted signatures were compared to those originally described in COSMIC using cosine similarity metrics. Manual curation, based on the expected biology for the cancer types in the study, was performed to refine the extracted signatures. For example, SBS4 (associated with tobacco exposure) was identified in multiple patients initially, but was not consistent with the expected biology for patients in the cohort with biliary tract cancer. Increasing the initial number of signatures revealed the presence of the platinum therapy-associated SBS = 31 in these patients.

### Copy number analyses

Log ratios and B-allele frequencies for the germline SNPs observed in the data were used to perform multisample segmentation, using a weighted version of the circular binary segmentation algorithm from DNACopy^55^. The resulting segments were used as input to PureCN^56^ to obtain allele-specific copy number, as well as estimates of tumor purity and ploidy. Initial PureCN solutions were manually checked and, for some cases, a different solution was used with the aim of maximizing similarity in copy number profiles between samples from the same patient. We used the purity estimates from PureCN to filter samples from the dataset: any sample with a purity estimate <40% was manually reviewed and removed if it had a non-aberrant copy number profile. Ten samples were removed using these criteria.

High-level amplifications were defined as events where the total number of copies in a segment was at least three times the estimated ploidy for a patient, whereas SCNAs resulting in a total copy number that was at least twice the estimated ploidy were defined as gains. Partial deletions were called if the observed number of copies was below half the estimated ploidy. Homozygous deletions and LOH (including copy number-neutral events) were classified separately. Proportions of trunk, branch, and private copy number alterations were determined by considering segments with altered copy number states. CIN was defined as the total fraction of the genome affected by any copy number event.

Driver copy number events were defined initially as high-level amplifications and homozygous deletions of oncogenes and tumor suppressor genes, respectively, as defined in the Cancer Gene Census. To avoid overestimating heterogeneity in copy number driver events, low-level gains in oncogenes were classified as driver events if at least one other sample from the same patient had a high-level amplification in the gene; this is analogous to the rescue method used for point mutations.

### Clonality and phylogenetic analyses

CCFs were computed only for somatic SNVs, as read counts for indels can be biased due to difficulties in alignment. As in ref. ^57^, CCF point estimates and CIs were derived by constructing a posterior distribution over a grid of 100 CCF values between 0 and 1, with probabilities determined by modeling observed reference and variant read counts with a binomial distribution. Tumor fraction estimates and copy number profiles from PureCN were used for the calculation. CCFs were only reported when the depth of coverage at a particular base was at least 30. Clonal mutations were defined as those with CCF ≥ 0.9, with the remaining mutations classified as subclonal.

We used a mutation’s multiplicity to determine whether it occurred early or late relative to a copy number event with major copy number ≥2 (ref. ^57^). In these regions, mutations with multiplicities >1 were assumed to have occurred before the SCNA. The presence of whole-genomic duplication was determined by considering the proportion of the genome with an even major copy number^58,59^. In general, WGD was said to have occurred if this proportion was >50%. Copy number profiles of samples were subsequently manually checked to confirm WGD.

Phylogenetic trees were constructed using the “pratchet” algorithm in Rphylip^60^. Visualizations were produced with ggtree. Both point mutations and copy number alterations were used when constructing trees.

### RNA analyses

Reads generated from RNA-seq were aligned with STAR^61^ to hg19 and feature counts were obtained with HTSeq. Raw counts were normalized and processed with DESeq2, and the variance-stabilizing transformation was applied to maintain homoskedasticity across the range of expression^62^. We used a collection of gene expressions signatures to profile the TME (Supplementary Table 4). These signatures were derived from analysis of gene expression data from The Cancer Genome Atlas as sets of genes, where the expression was highly correlated. To further ensure the signatures were related to the TME, we focused on signatures that had significantly higher scores than in breast and liver cell lines from the Cancer Cell Line Encyclopedia^63^. Scores were computed using single-sample GSEA^64^ and individual signature scores were transformed into *z*-scores across all the samples in the dataset. *T*-SNE^65^ to reduce the *z*-score matrices into two dimensions was performed using the R package “Rtnse” with perplexity = 10 and 5000 iterations. The MAD was used to summarize dispersion in signature *z*-scores.

### cfDNA analyses

Reads were aligned and processed as for the whole-exome data, and the rescue approach was used for some analyses when determining the proportions of trunk, branch, and private mutations detected (Figs. 3 and 4). All somatic point mutations with a VAF < 0.005 were filtered. Copy number profiles and tumor fraction in cfDNA were obtained running PureCN independently of the tissue samples, but possible PureCN solutions were manually reviewed, and the final profiles were selected to maintain relative consistency (ploidy estimates, gross segmentation profiles) with the tissue data.

For comparisons with tissue lesions (Figs. 3 and 4), mutation classification (i.e., trunk, branch, private) for mutations within the targeted panel was derived using the WES data tissue samples. Power calculations were performed using PureCN: assuming a false-positive rate of 5 × 10^−7^ and a sequencing error rate of 0.001 Mb^−1^, the minimum mutation CCF for which power of detection was least 90% in each sample^56,58^ was calculated. The median depth of coverage across loci at which mutations were found in tissue samples, but not in plasma was used when computing power. For each patient in Fig. 5, all possible combinations of metastases were considered when sampling between one and five lesions, and mean numbers of driver alterations identified were derived across these combinations. Within the sampling exercise, an alteration was classified as “truncal” if it was present in all lesions; for example, when sampling a random combination of four lesions, an alteration would be classified as truncal if it was observed in all four. ROC curves were constructed to evaluate the use of CCF thresholds in cfDNA, and the value nearest to the top-left corner of the curve was selected as the optimal threshold.

### Reporting summary

Further information on research design is available in the Nature Research Reporting Summary linked to this article.

## Supplementary information

Supplementary Information

Description of Additional Supplementary Files

Supplementary Data 1

Supplementary Data 2

Supplementary Data 3

Supplementary Data 4

Reporting Summary

## Data Availability

The sequencing data used in this study have been deposited in the European Genome-Phenome Archive under accession code EGAD00001007040. Uploaded data include BAM files containing raw, aligned sequencing data from the whole-exome, RNA-seq, and targeted cfDNA experiments. The data are available under restricted access with access controlled by the Termer Center Data Access Committee. The data may be accessed for research purposes by contacting juric.dejan@mgh.harvard.edu. In addition, Supplementary Dataset 1 contains sample-level statistics (e.g., mean depth of sequencing, tumor cellularity) used for CCF computations; Supplementary Dataset 2 contains point mutation calls for all samples; Supplementary Dataset 3 contains copy number segment calls for all samples; and Supplementary Dataset 4 contains the genes present in the gene expression signatures use to study the TME. Source data are provided with this paper.

## Source data

Source Data
