# Supplementary material for: Cell-free DNA captures tumor heterogeneity and driver alterations in rapid autopsies with pre-treated metastatic cancer
Source: Nat Commun. 2021 May 27;12:3199. doi: 10.1038/s41467-021-23394-4 (PMC8160338; doi:10.1038/s41467-021-23394-4)
Supplement: Supplementary file 1 — Supplementary Information [file 41467_2021_23394_MOESM1_ESM.pdf]

Supplementary Fig. 1

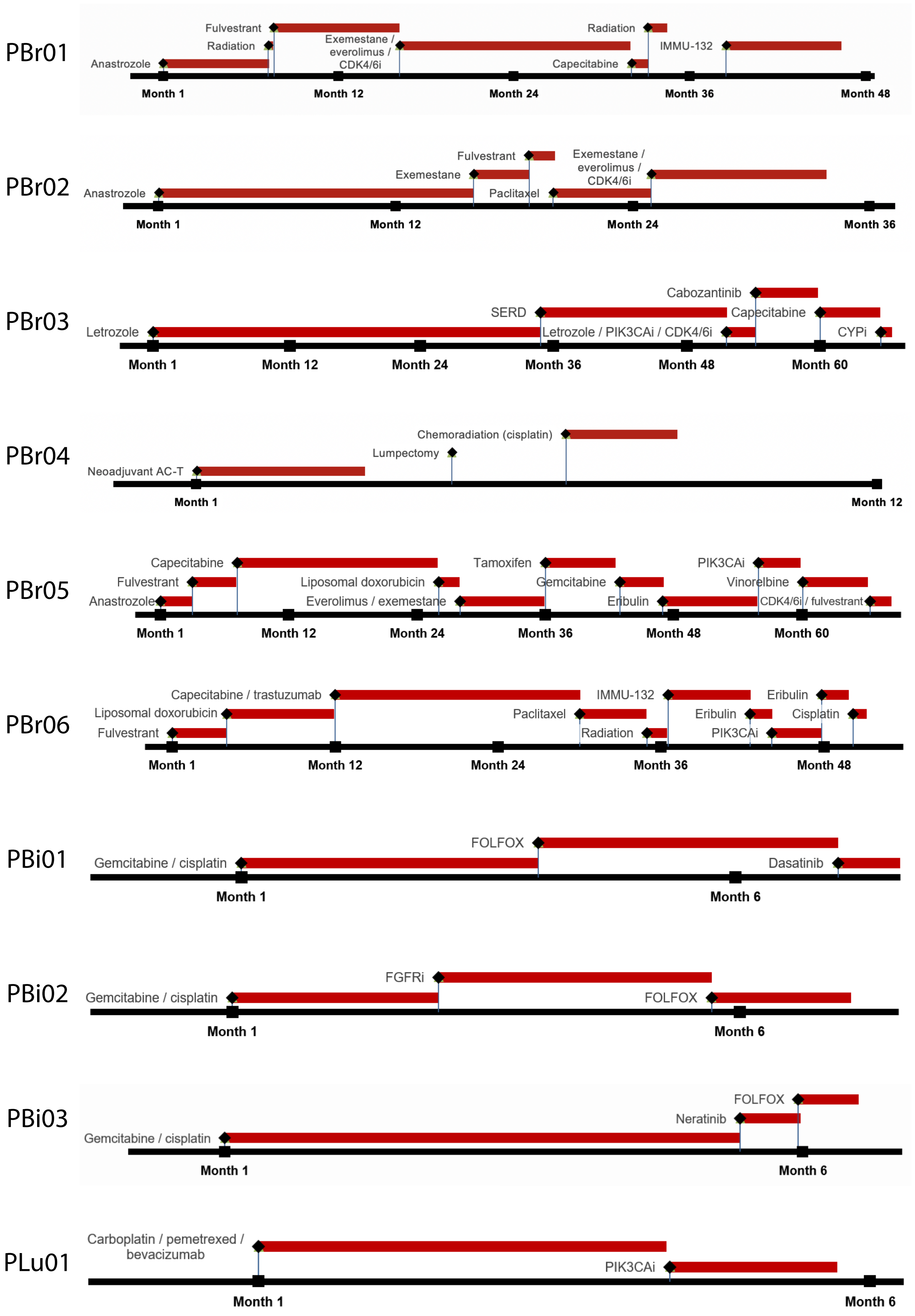

Supplementary Figure 1. Clinical histories of the rapid autopsy cases. Case histories, including treatments received, are presented for each patient.

Supplementary Fig. 2

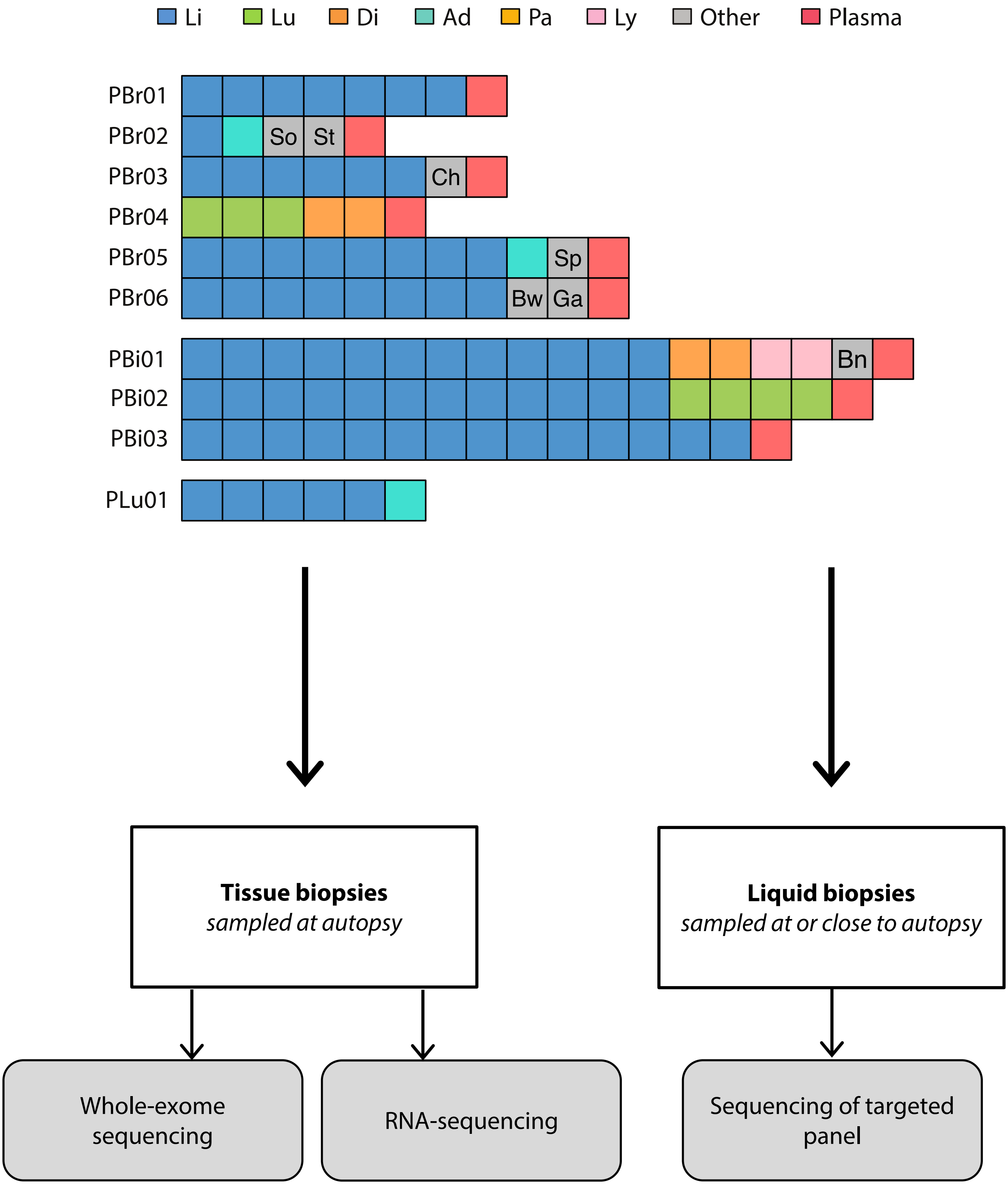

Supplementary Figure 2. Study overview. Lesions from spatially distinct metastatic lesions were profiled with whole-exome sequencing and RNA-sequencing. In addition, plasma samples were available for 9/10 patients at or near the time of autopsy. Complete anatomic descriptions are provided in ***Supplementary Dataset 1***.

Supplementary Fig. 3

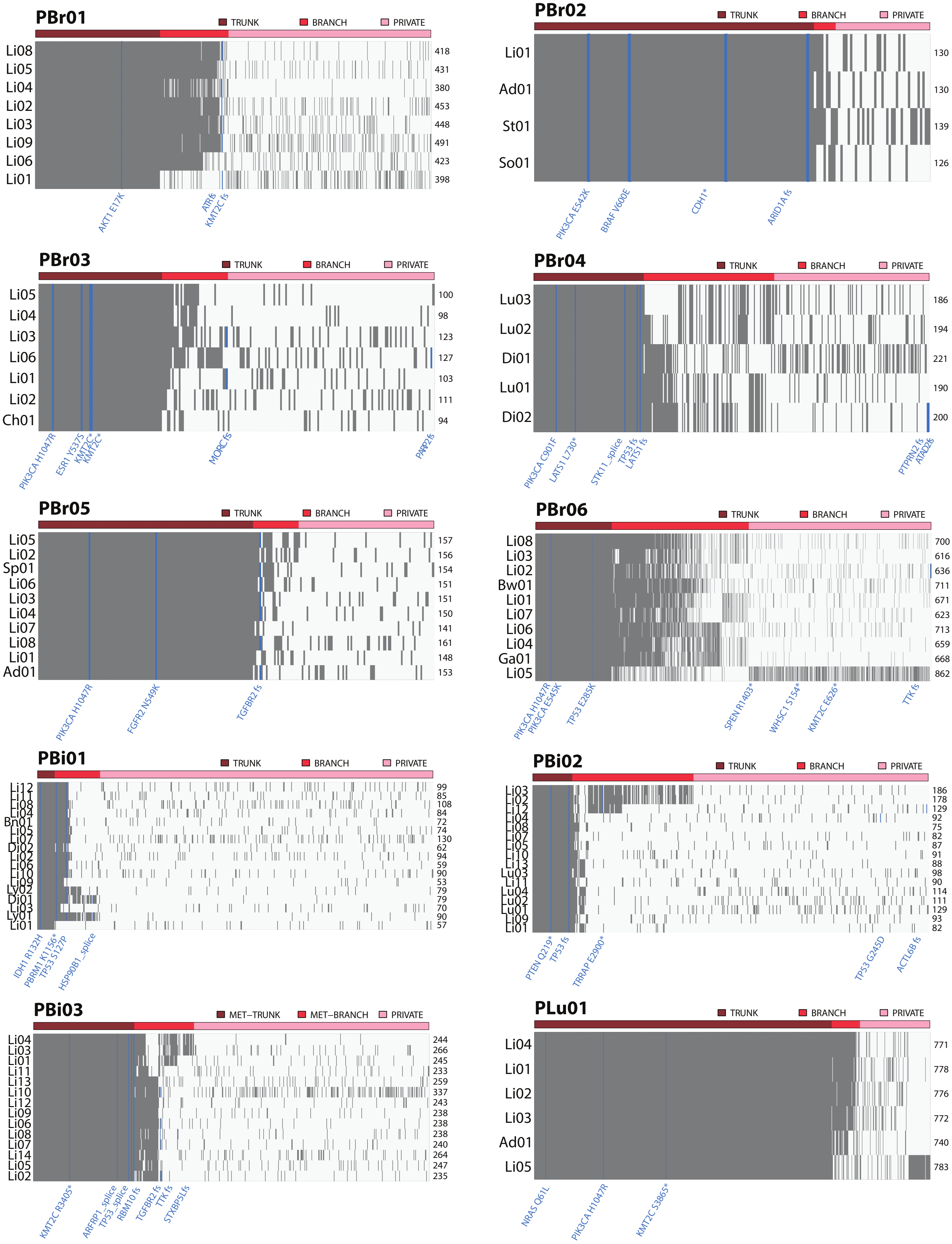

Supplementary Figure 3. Point mutations across samples from each patient.
Mutations likely to be functional ***(Methods)*** are indicated in blue. The proportions of trunk, branch and private mutations are shown above each mutation heatmap.

Supplementary Figure 4

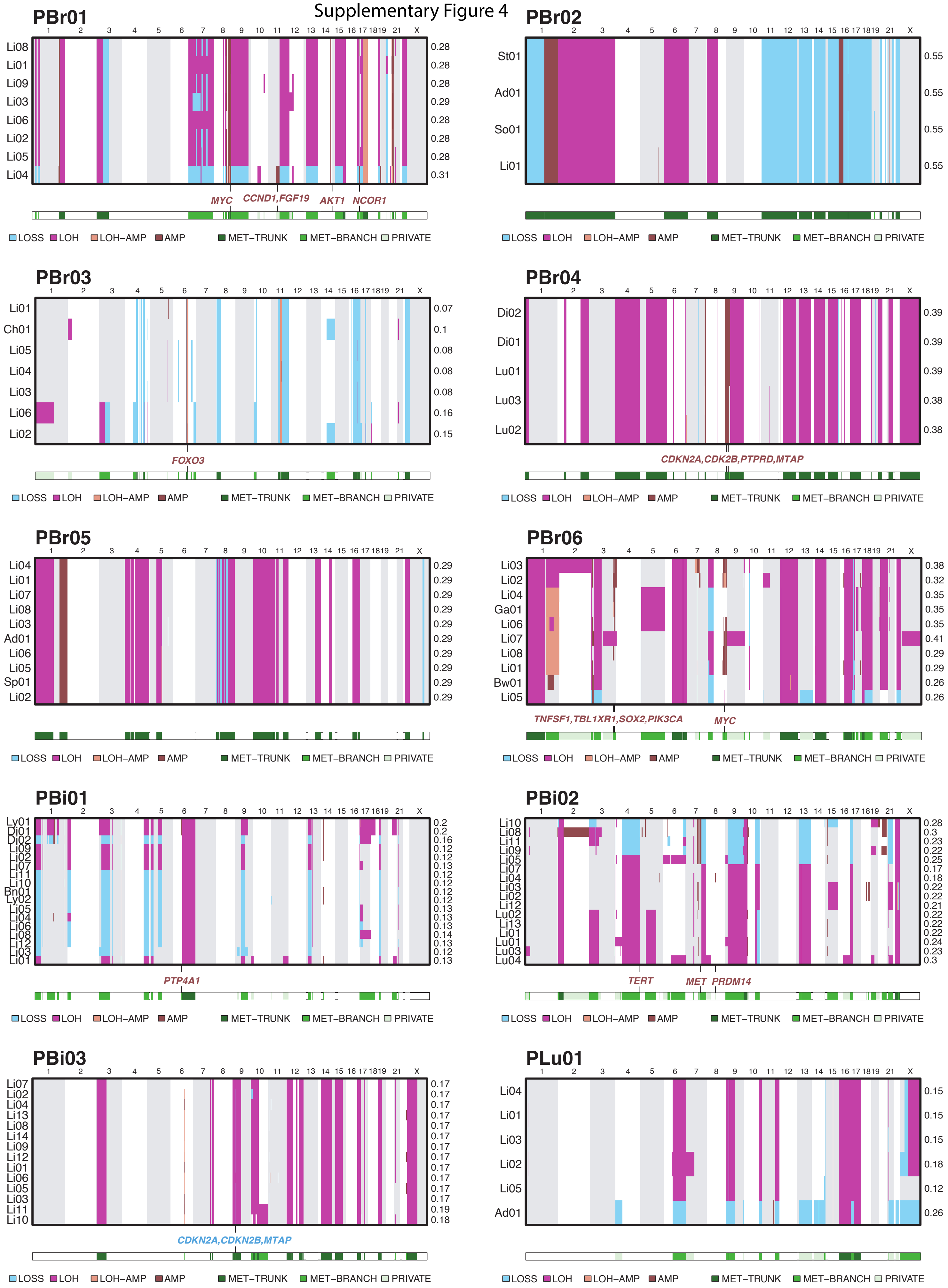

Supplementary Figure 4. Copy number profiles for samples from each patient. Genome-wide copy number changes are shown. Copy number changes likely to be functional are indicated. Copy number change classifications (i.e. trunk, branch, private) are shown below each heatmap.

Supplementary Fig. 5

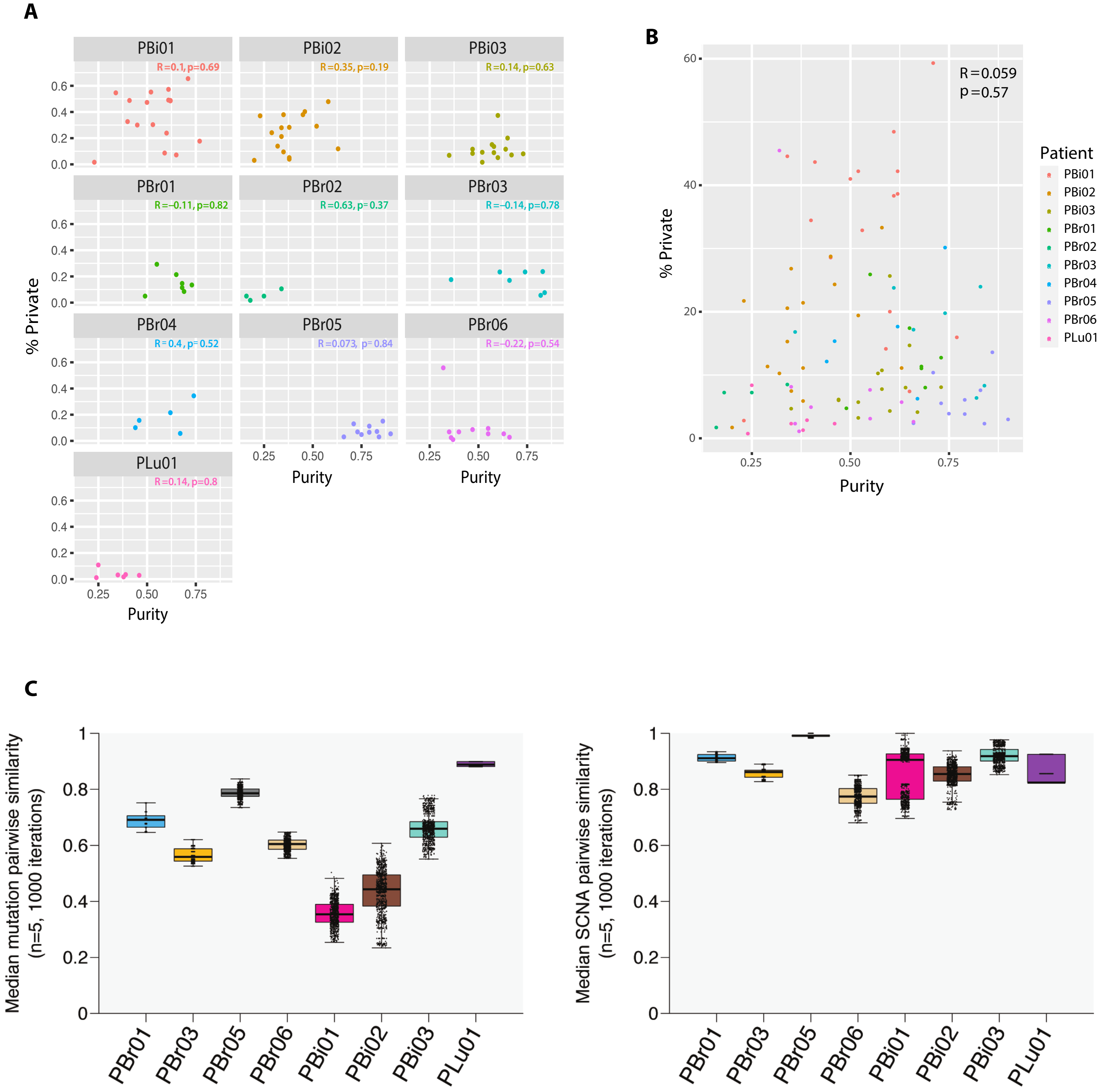

Supplementary Figure 5. Genetic heterogeneity between lesions from a patient. (A) Correlations between tumor cellularity and the percentage of private mutations for each patient in the cohort. R=Spearman correlation coefficient. (B) As in (A), but for the entire cohort together. (C) We randomly sampled 5 tissue samples from each patient with at least 5 lesions available and calculated the pairwise Jaccard similarity coefficient ***(Methods)*** to measure total mutation similarity between lesions. The sampling process was repeated 1000 times and the median similarity scores for each iteration are shown. For each patient, the boxplot's whiskers span the minimum and maximum value of the median similarity scores, the box spans the upper and lower quantiles, and the center represents the median of the distribution.

Supplementary Fig. 6

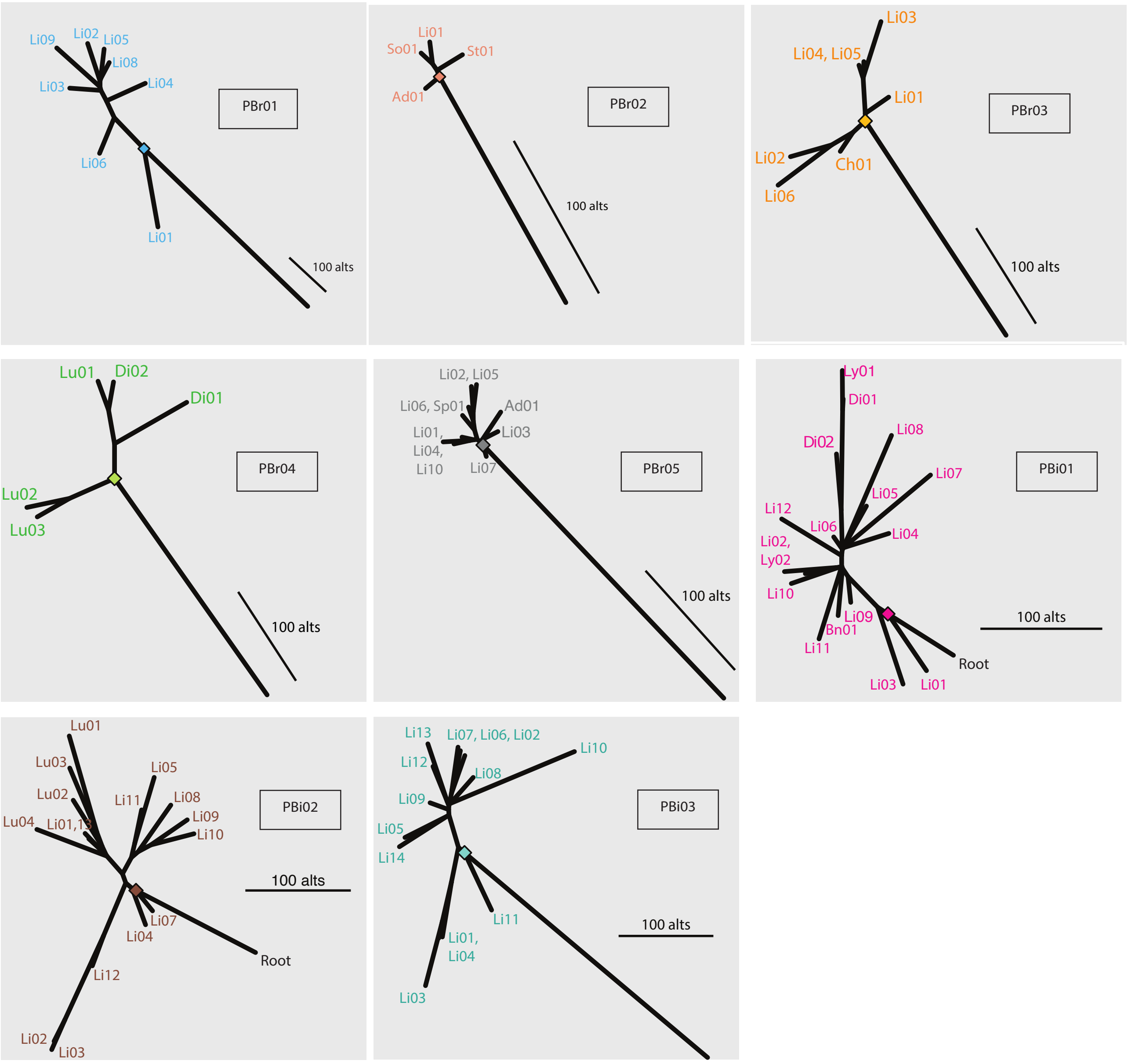

Supplementary Figure 6. Evolution of cancer metastases. Phylogenetic trees outlining the evolutionary relationships between lesions from each patient. Colored diamonds indicate the most recent common ancestor for all profiled lesions. The scale defines the number of somatic alterations on each branch, and includes both point mutations and altered copy number segments.

Supplementary Fig. 7

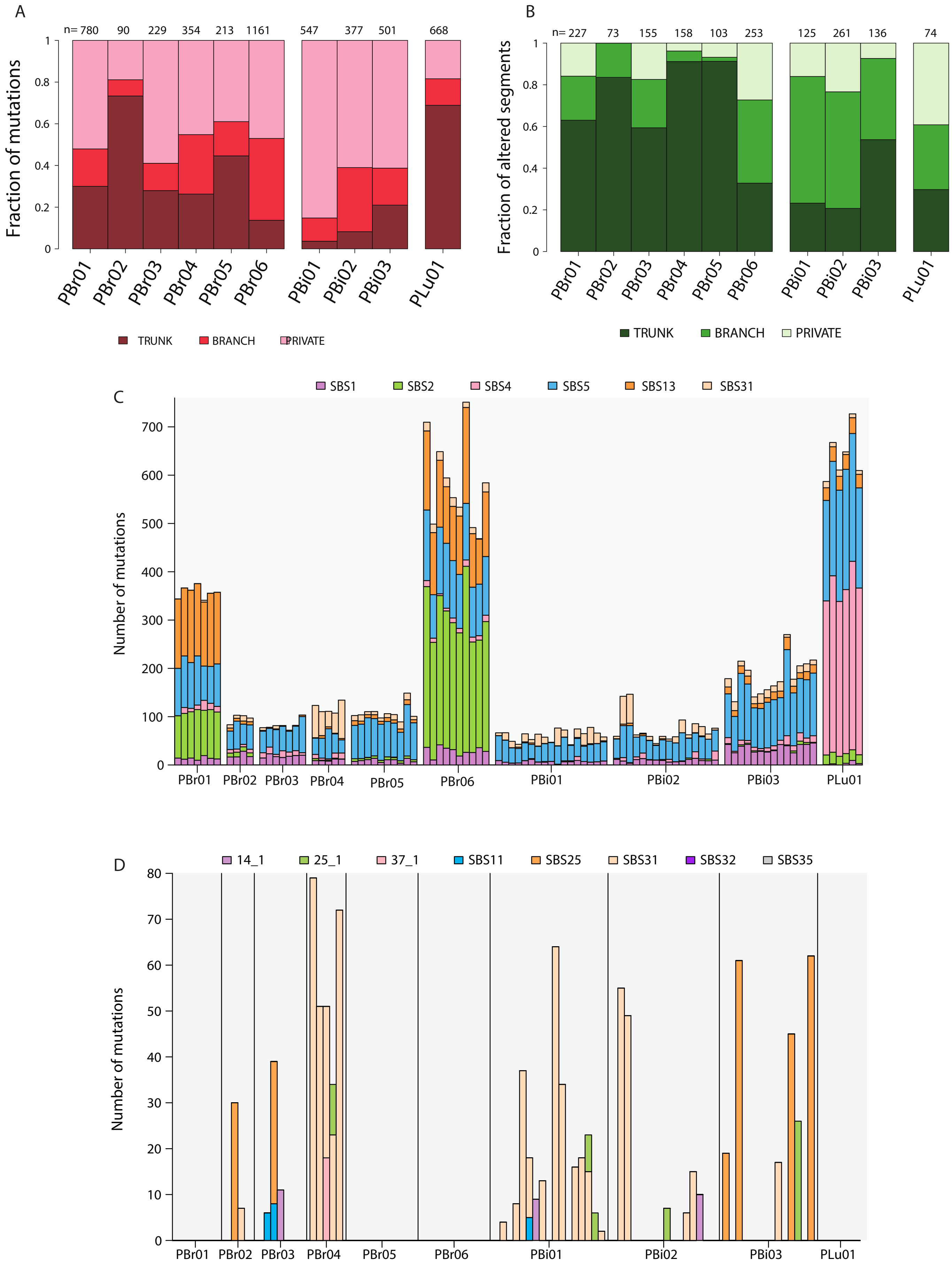

Supplementary Figure 7. Single-base substitution mutation signatures in the rapid autopsy cohort. (A) Proportions of truncal, branch and point mutations identified in each patient in the cohort. The numbers of all mutations found in any lesion from a patient are indicated above the bars. (B) As for (A), but for altered copy number segments. (C) Mutation signatures identified in all lesions in the dataset. Each bar represents an individual tissue sample, samples are grouped by patient, and colors represent the mutation signatures from COSMIC (single-base substitutions; SBS) that were most similar to the six mutation signatures observed across the autopsy cohort. Signatures are defined in ***Figure 1F***. (D) Numbers of therapy-related mutations identified across all samples grouped by patient. Mutation signatures were curated from COSMIC and from *Pich et al.* (*23*). Colors indicate the following signatures: Pich_14_1 (associated with cisplatin/oxaliplatin exposure), Pich_25_1 (carboplatin), Pich_37_1 (oxaliplatin), SBS11 (temozolomide), SBS25 (general chemotherapy), SBS31(carboplatin/cisplatin), SBS32 (azathioprine), SBS35 (platinum).

Supplementary Fig. 8

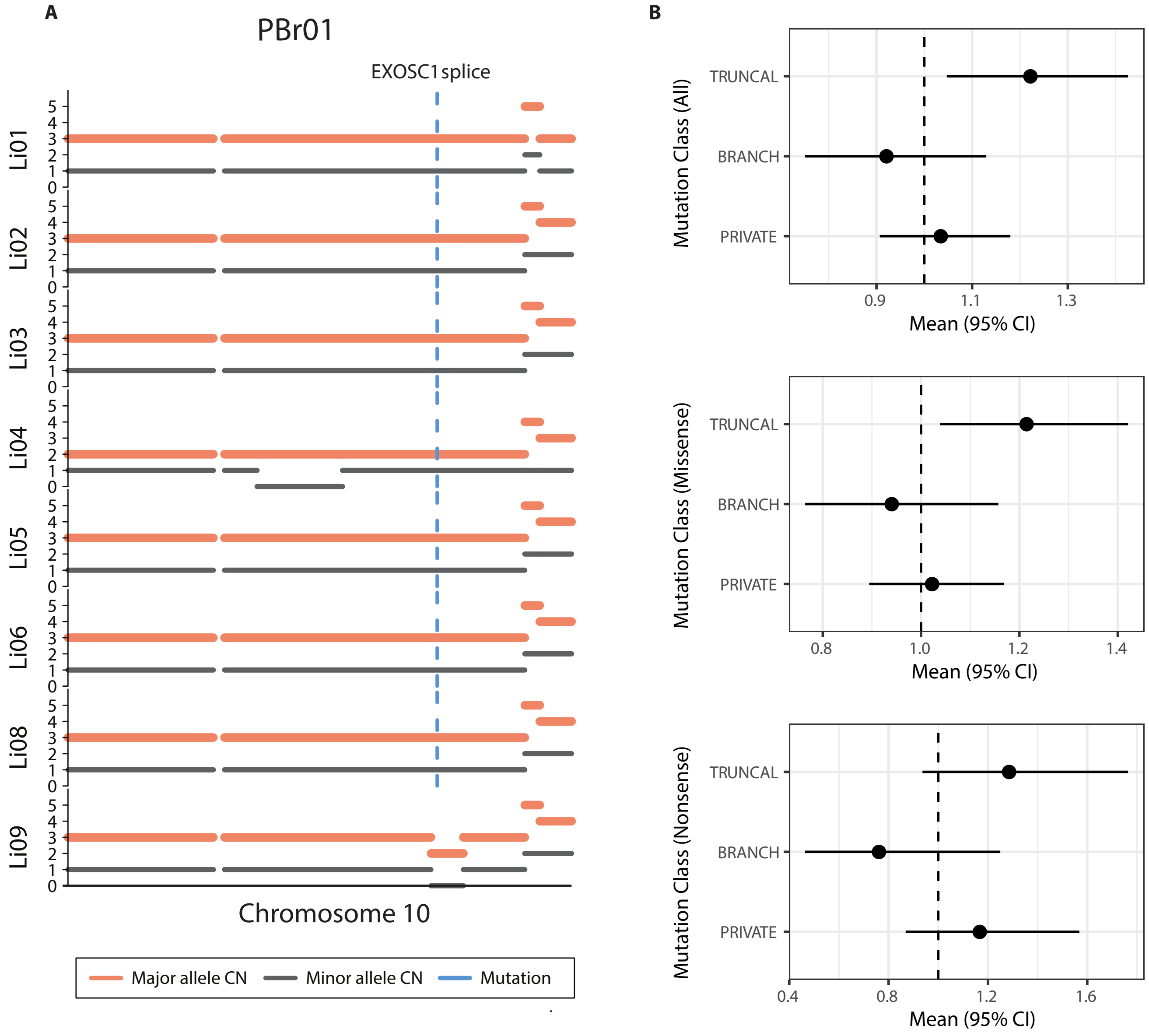

Supplementary Figure 8. Mutation loss by LOH. (A) Allele-specific copy number data can provide evidence for loss of mutant alleles by SCNAs. Here, the *EXOSC1* splice-site mutation on Chromosome 10 was identified in all lesions in PBr01 except for Li09. LOH with loss of the minor allele was evident at the gene locus in this lesion. The data together suggest that the *EXOSC1* mutant allele may have been lost in this lesion. (B) dN/dS estimates for mutations across the autopsy cohort. Estimates for specific mutation subsets are shown (all mutations; missense mutations; nonsense mutations). Mutations are also classified by class (trunk, branch, private). Points show point estimates for each category, dashed lines highlight dN/dS=1, and solid lines depict 95% confidence intervals (CI) for the estimates.

Supplementary Fig. 9

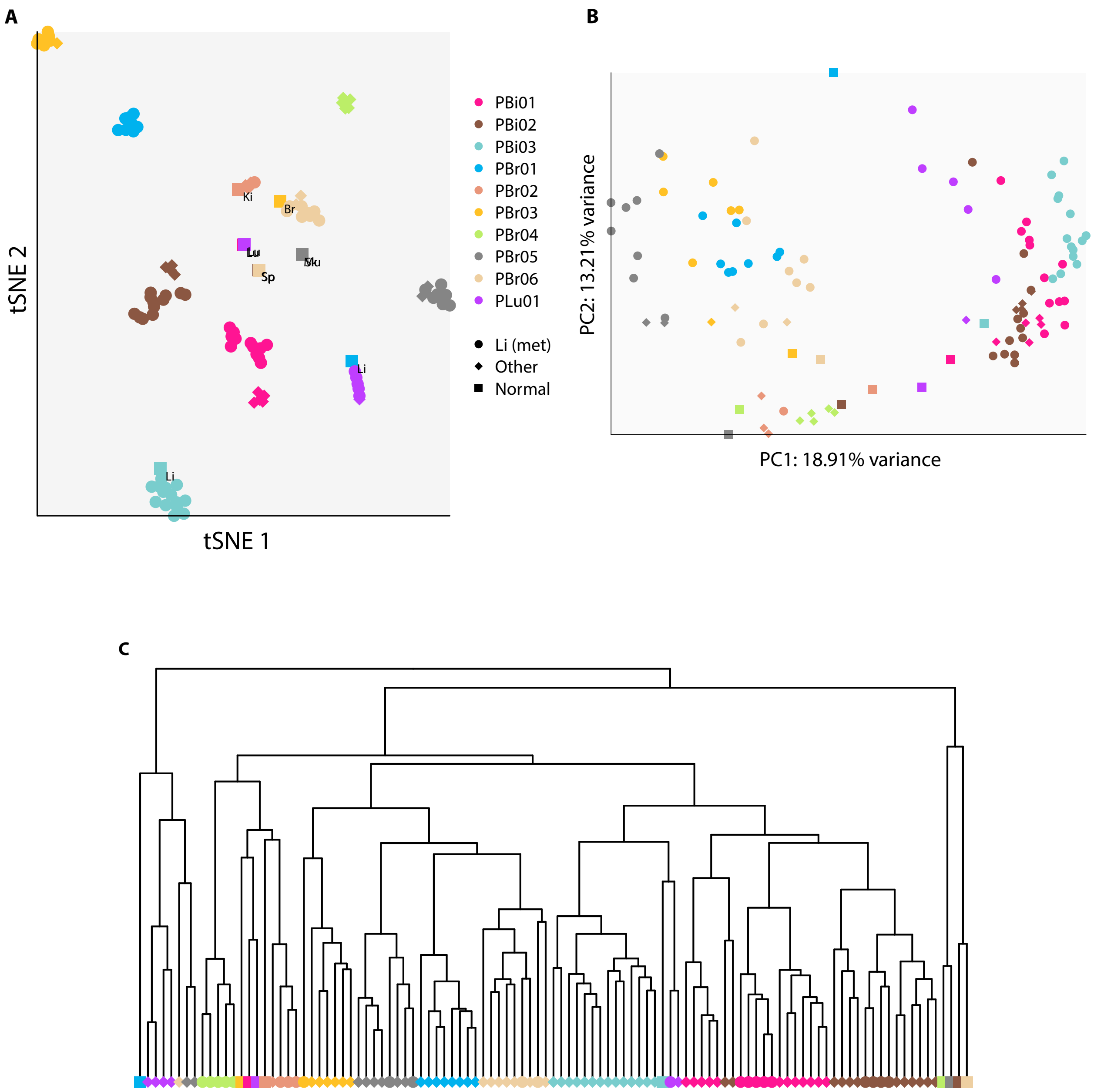

Supplementary Figure 9. Contributions of immune and stromal cells to observed tumor cellularity. (A) t-SNE analysis was performed across all samples using whole transcriptome data. The figure shows clusters obtained (t-SNE perplexity = 5). Colors indicate patients and shapes indicate sample type (Li=liver lesion, Normal=histologically normal tissue). Two-letter codes indicate the normal tissue of origin: Ki=kidney, Br=breast, Lu=lung, Sp=spleen, Li=liver, Sk=skeletal muscle, Mu=muscle. (B) Principal component analysis performed across all samples using the whole transcriptome. The percentages of variance explained by the first two principal components are indicated on the axes. Colors and points can be interpreted using the legend from (A). (C) Dendrogram from unbiased hierarchical clustering (based on Euclidean distance) of all samples. Colors are defined by patient as shown in (A). Shapes indicate whether a specific lesion is from the liver (circle), from another site (diamond), or from histologically normal tissue (square). Colors indicate patients as defined in the legend for part ***(A)***.

Supplementary Fig. 10

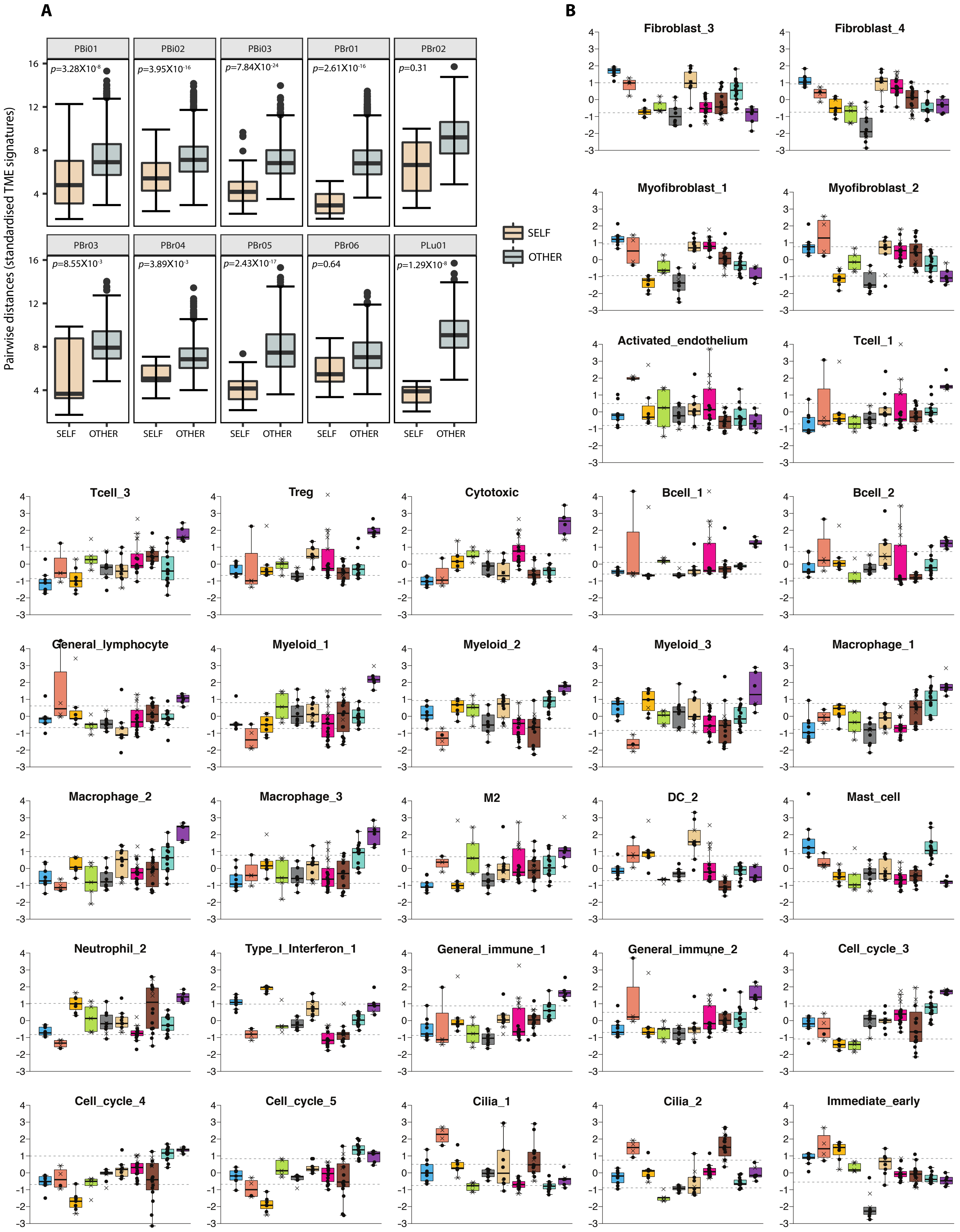

Supplementary Figure 10. TME-associated gene expression signatures. (A) Gene expression signature scores were derived as described in ***Methods*** and Z-score transformed. For each patient, Euclidean distances based on the scores matrix were computed between all tumor samples from that patient ('SELF') or between tumor samples from that patient and all other patients ('OTHER'). The boxplots illustrate the distributions of Euclidean distances: the whiskers indicate the maxima and the minima for the specified categories, the boxes indicate the upper and lower quartiles, and the center depicts the median. Outlier points are shown separately. Indicated *p*-values were derived for each patient using Wilcoxon tests comparing all pairwise 'SELF' distances with all pairwise 'OTHER' distances. (B) Z-scores of gene expression signatures are shown for metastatic lesions each patient. Lesion type (liver or non-liver) is indicated by point shapes. All colors and point shapes correspond to those in ***Figure 3*** and ***Supplementary Figure 9***. For all boxplots, whiskers span the minimum and maximum value of the variable distribution (distances or expression levels), the box spans the upper and lower quantiles, and the center represents the median of the distribution.

Supplementary Fig. 11

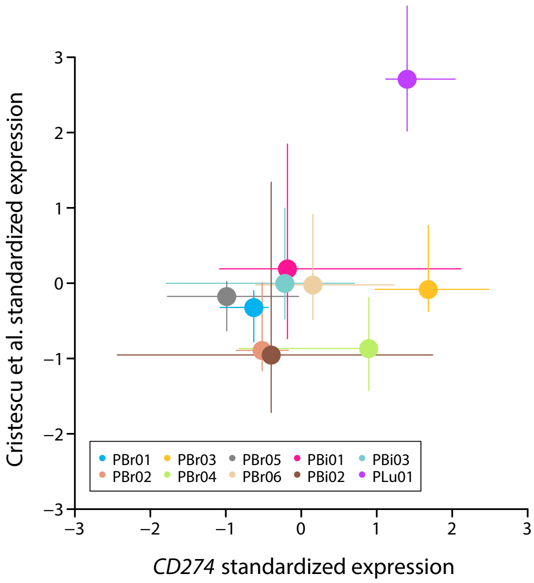

Supplementary Figure 11. Correlations between TME-associated signatures. Points represent median *CD274* and *Cristescu et al.* z-scores for each patient and the ranges of scores (x-axis=range of *CD274* expression, y-axis=range of *Cristescu et al.* expression) for the patients are indicated by lines.

Supplementary Fig. 12

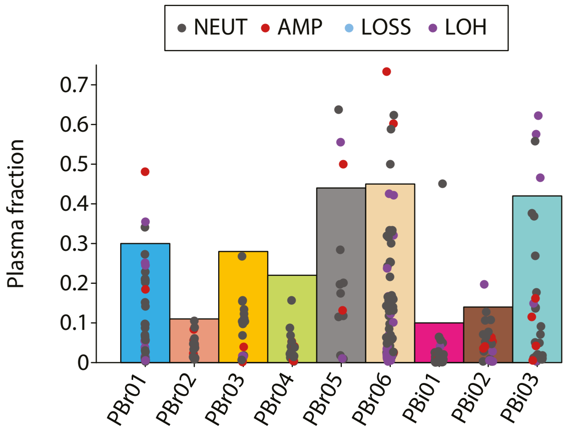

Supplementary Figure 12. Estimated fractions of tumor DNA in plasma samples. PureCN-derived estimates of tumor fractions in plasma are shown for each patient. Points indicate the raw variant allele fractions observed in the indicated copy number classes.

Supplementary Fig. 13

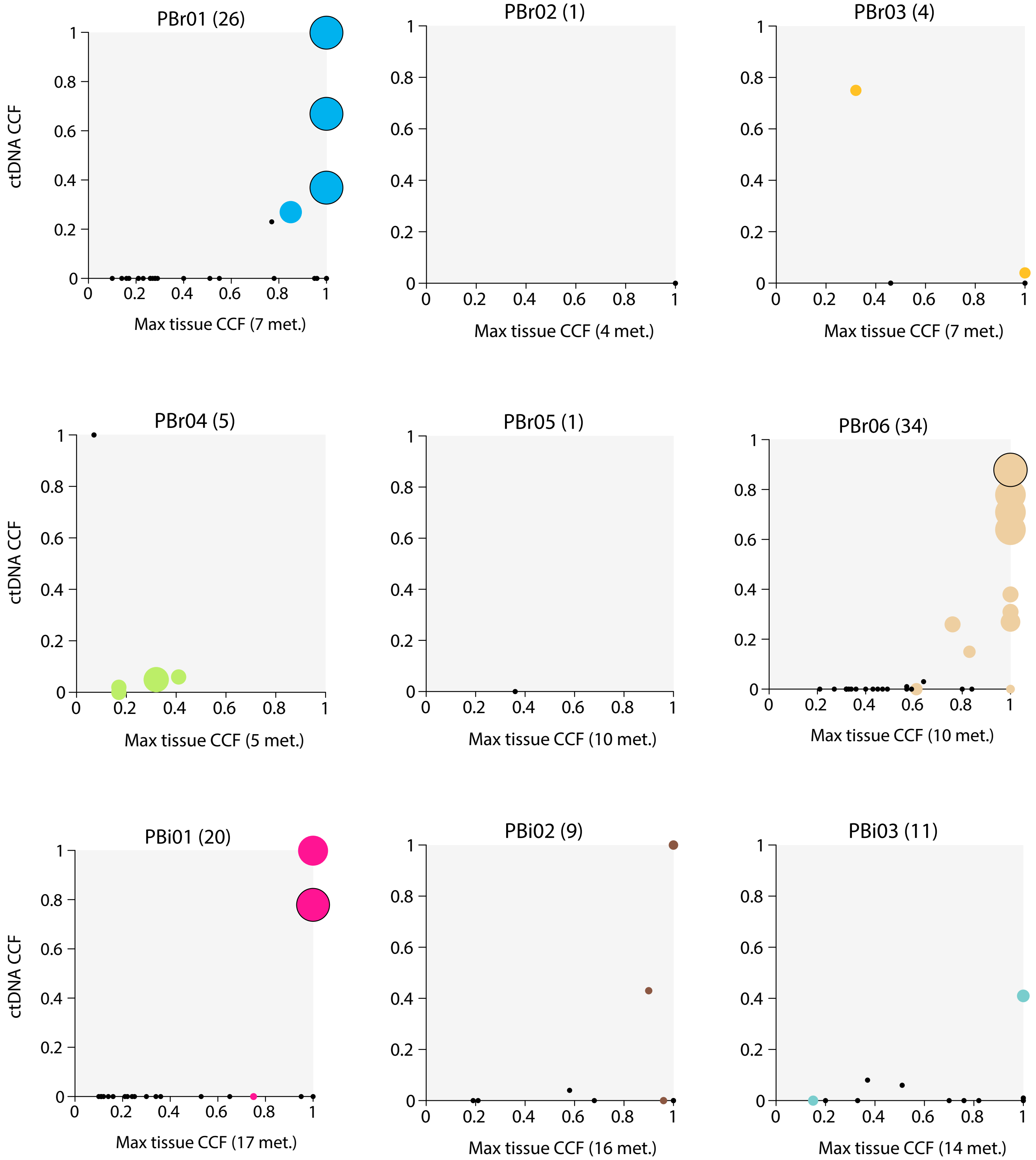

Supplementary Figure 13. Correlations between lesion count and mutation CCF in cfDNA. As in ***Figure 4C***, each circle depicts a non-truncal mutation, and the size of the circle represents the number of lesions in which the mutation was found. The maximum CCF observed for the mutation across all tissue samples, and the ctDNA CCF for the mutation observed in cfDNA are shown. The number of non-truncal mutations in each case is shown in brackets.

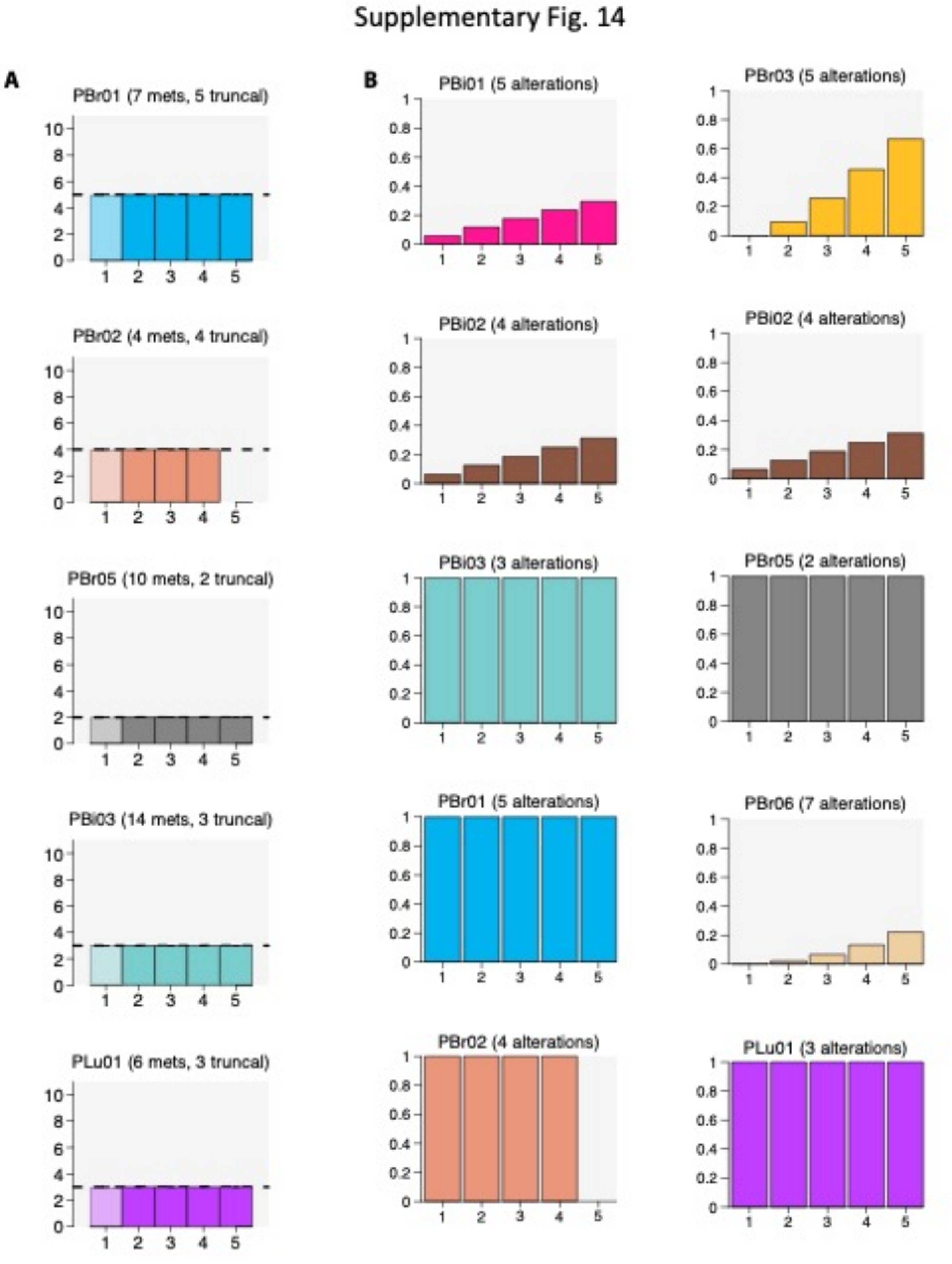

Supplementary Figure 14. Probabilities of identifying all driver alterations by repeated sampling. (A) As for ***Figure 5A***, but showing patients where all mutations were truncal. (B) The proportions of sampled combinations where all driver alterations are identified when sampling all possible 1-5 tissue sample combinations for each patient. All driver alterations in PBr04 and PLu01 were truncal. The total number of observed driver alterations (mutations and SCNAs) is shown in brackets.

Supplementary Fig. 15

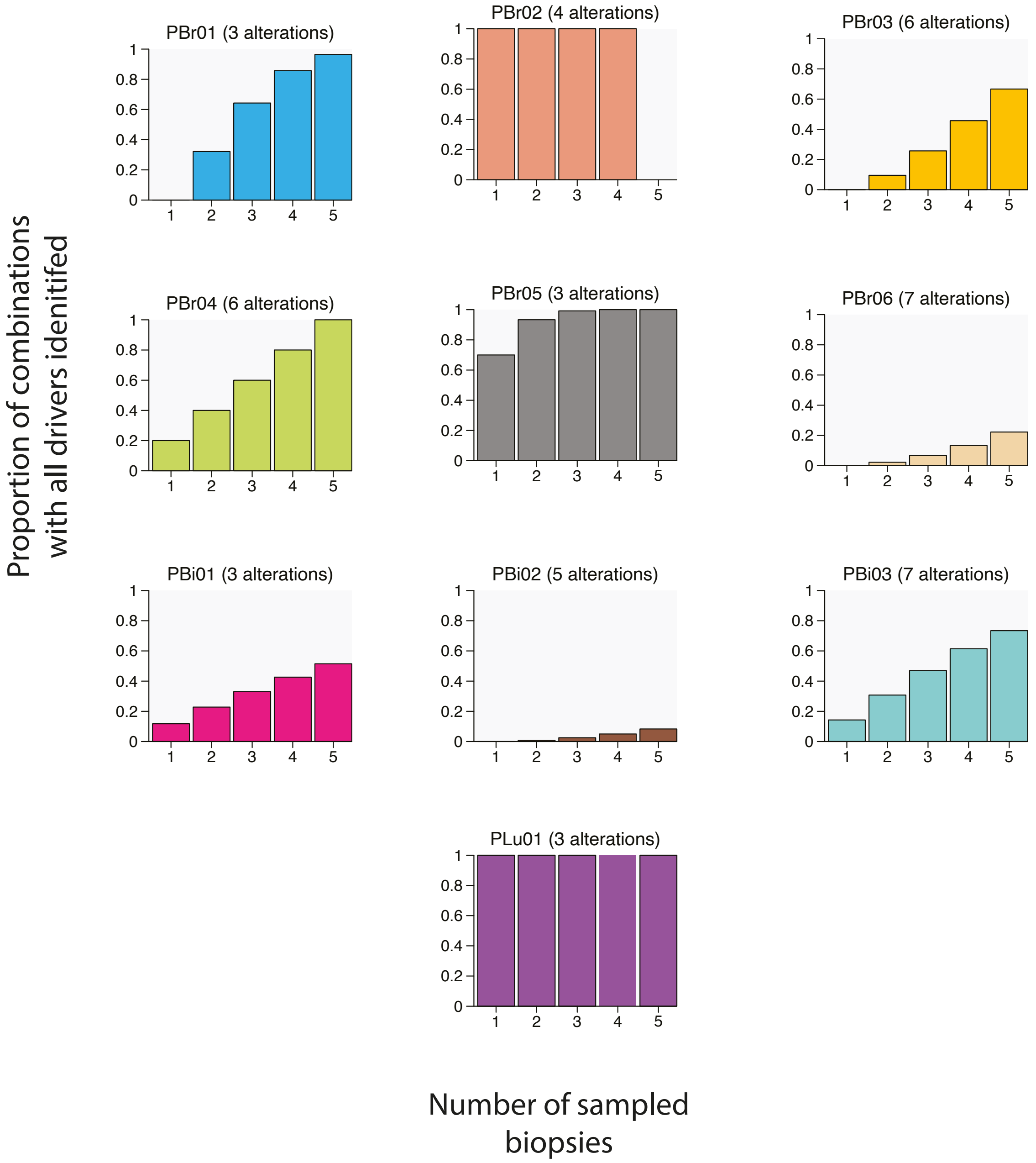

Supplementary Figure 15. Assessment of genetic heterogeneity with separate driver alteration list. Analyses were repeated focusing on any non-silent alteration in a previously defined list of driver genes (*22*). Only somatic point mutations were considered, as driver SCNAs were not defined in the original scheme. As in ***Supplementary Figure 14B***, the proportions of all driver mutations identified upon sampling all possible 1-5 tissue sample combinations are shown.

Supplementary Fig. 16

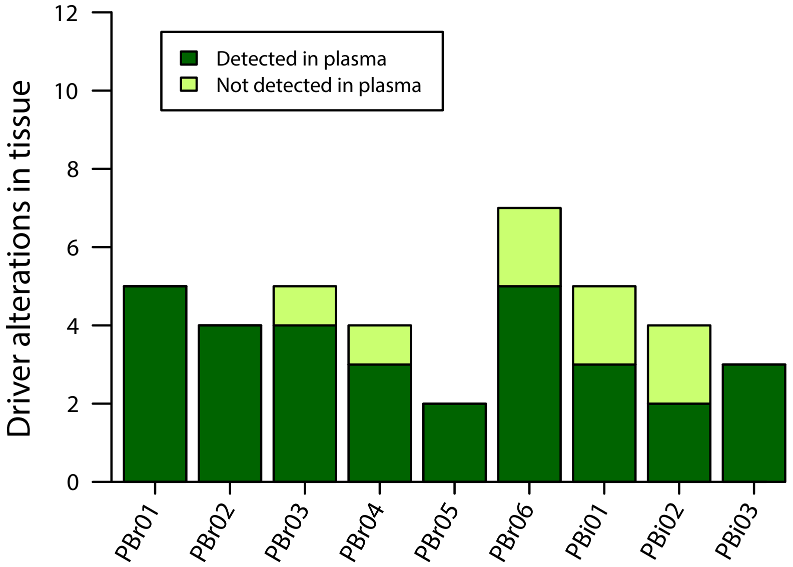

Supplementary Figure 16. Driver alteration detection in a plasma sample. The numbers of driver alterations identified in cfDNA are shown for the nine patients with a plasma sample available.

Supplementary Fig. 17

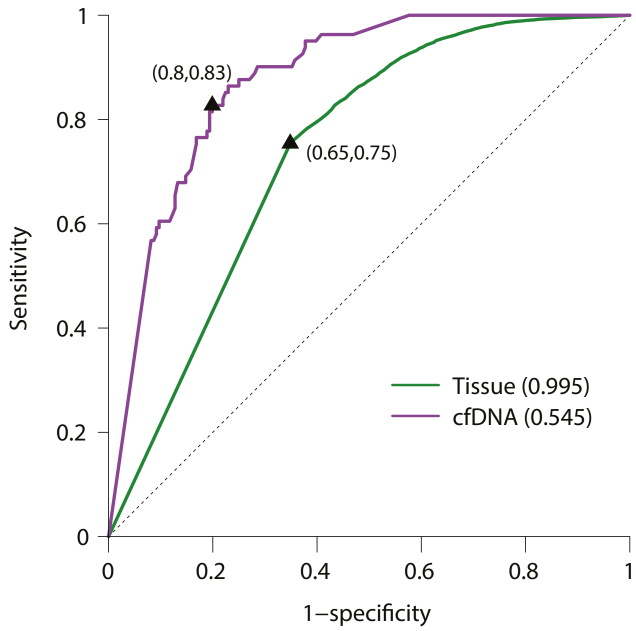

Supplementary Figure 17. ROC analysis for identifying CCF thresholds. Lines show sensitivity and specificity of classifying mutations as 'truncal' or 'non-truncal' based on mutation CCF in tissue or ctDNA. The coordinates indicated show the sensitivity and

specificity obtained at the optimal thresholds (points closest to the top-left corner) for either all tissue samples (CCF=0.995) or all cfDNA samples (ctDNA CF=0.545).

Patient	CT Scan Details	Time of cfDNA sampling
PBi01	23 days prior to death (CT chest / abdomen / pelvis): ~15-20 scattered pulmonary nodules, up to 4 mm; ascites; >30 confluent hepatic lesions, up to 13 cm; 2 bone lesions (rib, acetabulum); 5-10 increased lymph nodes (perihepatic, portacaval, pericardiophrenic, up to 3.1 cm)	At autopsy
PBi02	39 days prior to death (CT abdomen / pelvis), 30 days prior to death (CT chest): >50 confluent liver lesions, individually up to 4.5 cm; 5-10 peripancreatic, gastrohepatic, mesenteric lymph nodes up to 1.4 cm; 5-10 scattered pulmonary nodules, up to 9 mm	At autopsy
PBi03	48 days prior to death (chest CT), 37 days prior to death (abdominal/pelvis CT): >40 scattered and confluent hepatic mets, up to 5.5 cm; ascites; 5-10 scattered pulmonary nodules, up to 5 mm; scattered mediastinal lymph nodes, up to 13 mm	42 days prior to autopsy
PBr01	16 days prior to death (CT chest / abdomen / pelvis): >40 scattered and confluent hepatic metastases, up to 3.5 cm; small volume ascites; single 2 mm pulmonary nodule of unclear significance; sclerotic bone mets involving bilateral femurs, pelvic bones, sacrum, and lumbar spine.	At autopsy
PBr02	25 days prior to death (CT/PET), 18 days prior to death (chest CT), 17 days prior to death (abdomen CT): ascites; poorly defined retroperitoneal soft tissue mass, at least 2.6 cm in length; diffuse skeletal involvement involving calvarium, ribs, thoracolumbar spine, pelvis	4 days prior to autopsy
PBr03	34 days prior to death (CT chest / abdomen / pelvis): >20 scattered liver lesions, up to 1.8 cm; soft tissue mass next to the service, 2.7 cm; diffuse bony involvement, including calvarium, cervical and thoracolumbar spine, ribs, pelvis, bilateral upper and lower extremities\	3 days prior to autopsy
PBr04	30 days prior to death (abdomen / pelvis CT); 9 days prior to death (chest CT): 4.1 cm right upper lobe lung mass; 5-10 scattered pulmonary nodules, up to 6 mm; pleural effusion; multiple mediastinal, hilar, supraclavicular lymph nodes, up to 5.0 cm	4 days prior to autopsy
PBr05	59 days prior to death (CT-PE), 46 days prior to death (abdomen / pelvis CT): >30 scattered and confluent liver lesions, up to 3.2 cm; 20-30 scattered pulmonary nodules, up to 4mm	30 days prior to autopsy
PBr06	50 days prior to death (CT chest / abdomen / pelvis): >50 scattered and confluent liver lesions, largest coalesced mass 5.5 cm; hazy abdominal soft tissue around R portal vein; multiple bony lesions involving thoracic vertebrae, ribs, scapula, manubrium	At autopsy
PLu01	13 days prior to death (CT chest / abdomen/pelvis): 10-20 scattered and confluent liver mets, up to 5.5 cm; bilateral adrenal lesions, up to 4.4 cm; scattered bone lesions in sacrum and iliac bones; 10-20 scattered pulmonary nodules, up to 9 mm	cfDNA not available

Supplementary Table 1 – Radiological scans of lesions in the ten cases prior to death. Anatomic sites and lesion sizes are shown where possible. In addition, the time at which the CT scan was obtained (relative to time of autopsy) is also indicated. Similarly, the time at which liquid biopsy relative to autopsy was sampled is also shown.
