# Supplementary material for: Cell-free DNA captures tumor heterogeneity and driver alterations in rapid autopsies with pre-treated metastatic cancer
Source: Nat Commun. 2021 May 27;12:3199. doi: 10.1038/s41467-021-23394-4 (PMC8160338; doi:10.1038/s41467-021-23394-4)
Supplement: Supplementary file 2 — Description of Additional Supplementary Files [file 41467_2021_23394_MOESM2_ESM.pdf]

Description of Additional Supplementary Files

File Name: Supplementary Data 1
Description: **Overview of samples from each patient.** Precise sample location and sequencing metrics are indicated for each sample. Tumor mutation burden, tumor cellularity, and chromosomal instability estimates are also shown.

File Name: Supplementary Data 2
Description: **Point mutation calls for all samples.**

File Name: Supplementary Data 3
Description: **Copy number segments estimated with PureCN.**

File Name: Supplementary Data 4
Description: **Definitions of gene expression signatures used to profile the TME.**
