# Supplementary material for: Cell-free DNA captures tumor heterogeneity and driver alterations in rapid autopsies with pre-treated metastatic cancer
Source: Nat Commun. 2021 May 27;12:3199. doi: 10.1038/s41467-021-23394-4 (PMC8160338; doi:10.1038/s41467-021-23394-4)
Supplement: Supplementary file 7 — Reporting Summary [file 41467_2021_23394_MOESM7_ESM.pdf]

nature research

Corresponding author(s): Dejan Juric, Catarina D. Campbell

Last updated by author(s): Feb 24, 2021

Reporting Summary

Nature Research wishes to improve the reproducibility of the work that we publish. This form provides structure for consistency and transparency in reporting. For further information on Nature Research policies, see our Editorial Policies and the Editorial Policy Checklist.

Statistics

For all statistical analyses, confirm that the following items are present in the figure legend, table legend, main text, or Methods section.

n/a	Confirmed	
☐	☒	The exact sample size (n) for each experimental group/condition, given as a discrete number and unit of measurement
☐	☒	A statement on whether measurements were taken from distinct samples or whether the same sample was measured repeatedly
☐	☒	The statistical test(s) used AND whether they are one- or two-sided<br>*Only common tests should be described solely by name; describe more complex techniques in the Methods section.*
☐	☒	A description of all covariates tested
☒	☐	A description of any assumptions or corrections, such as tests of normality and adjustment for multiple comparisons
☐	☒	A full description of the statistical parameters including central tendency (e.g. means) or other basic estimates (e.g. regression coefficient) AND variation (e.g. standard deviation) or associated estimates of uncertainty (e.g. confidence intervals)
☐	☒	For null hypothesis testing, the test statistic (e.g. F, t, r) with confidence intervals, effect sizes, degrees of freedom and P value noted<br>*Give P values as exact values whenever suitable.*
☒	☐	For Bayesian analysis, information on the choice of priors and Markov chain Monte Carlo settings
☒	☐	For hierarchical and complex designs, identification of the appropriate level for tests and full reporting of outcomes
☐	☒	Estimates of effect sizes (e.g. Cohen's d, Pearson's r), indicating how they were calculated

Our web collection on statistics for biologists contains articles on many of the points above.

Software and code

Policy information about availability of computer code

Data collection	No software was used.
Data analysis	Only open-source were used in these analyses. Primary software used included BWA, the GATK suite, Mutect, Pindel, STAR, HTSeq. References for all are provided, and more details are provided in the Methods section.

For manuscripts utilizing custom algorithms or software that are central to the research but not yet described in published literature, software must be made available to editors and reviewers. We strongly encourage code deposition in a community repository (e.g. GitHub). See the Nature Research guidelines for submitting code & software for further information.

Data

Policy information about availability of data

All manuscripts must include a data availability statement. This statement should provide the following information, where applicable:

- Accession codes, unique identifiers, or web links for publicly available datasets
- A list of figures that have associated raw data
- A description of any restrictions on data availability

The sequencing data used in this study have been deposited in the European Genome-Phenome Archive under accession code EGAD00001007040 (https://ega-archive.org/datasets/EGAD00001007040). The data are available under restricted access as described in the data access policy associated with the accession code.

Source data are provided with this paper are available in the Source Data file. In addition, Supplementary Dataset 1 contains sample-level statistics (e.g. mean depth of sequencing, tumor cellularity) used for CCF computations; Supplementary Dataset 2 contains point mutation calls for all samples; Supplementary Dataset 3 contains copy number segment calls for all samples; and Supplementary Dataset 4 contains the genes present in the gene expression signatures use to study the TME.

Field-specific reporting

Please select the one below that is the best fit for your research. If you are not sure, read the appropriate sections before making your selection.

- [x] Life sciences
- [ ] Behavioural & social sciences
- [ ] Ecological, evolutionary & environmental sciences

For a reference copy of the document with all sections, see nature.com/documents/nr-reporting-summary-flat.pdf

Life sciences study design

All studies must disclose on these points even when the disclosure is negative.

Sample size	No sample-size calculations were performed. Sample count reflects the number of patients giving consent to the rapid autopsy procedure.
Data exclusions	Samples with very low tumor purity (estimated by gene expression and/or copy number analysis) were removed from the study. In addition, one sample with uncertain identity was removed (genotypes did not match genotypes in other samples from the same patient).
Replication	No replication was performed.
Randomization	Not relevant as unbiased comparisons between subsets of samples/patients were not performed.
Blinding	Not relevant as unbiased comparisons between subsets of samples/patients were not performed.

Reporting for specific materials, systems and methods

We require information from authors about some types of materials, experimental systems and methods used in many studies. Here, indicate whether each material, system or method listed is relevant to your study. If you are not sure if a list item applies to your research, read the appropriate section before selecting a response.

Materials & experimental systems

n/a	Involved in the study	
☒	☐	Antibodies
☒	☐	Eukaryotic cell lines
☒	☐	Palaeontology and archaeology
☒	☐	Animals and other organisms
☐	☒	Human research participants
☒	☐	Clinical data
☒	☐	Dual use research of concern

Methods

n/a	Involved in the study	
☒	☐	ChIP-seq
☒	☐	Flow cytometry
☒	☐	MRI-based neuroimaging

Human research participants

Policy information about studies involving human research participants

Population characteristics	All participants are patients diagnosed with advanced metastatic cancer before passing away. 6 patients had breast cancer; 3 had cholangiocarcinomas; 1 had lung cancer. Different treatments were used depending on medical need (Supplementary Figure 1). Patient demographics (age, sex etc.) varied are described in Table 1.
Recruitment	Patients with advanced cancers.
Ethics oversight	The Partners Human Research Committee

Note that full information on the approval of the study protocol must also be provided in the manuscript.
